# Rapid Bladder Interleukin-10 Synthesis in Response to Uropathogenic Escherichia coli Is Part of a Defense Strategy Triggered by the Major Bacterial Flagellar Filament FliC and Contingent on TLR5

**DOI:** 10.1128/mSphere.00545-19

**Published:** 2019-11-27

**Authors:** Dhruba Acharya, Matthew J. Sullivan, Benjamin L. Duell, Kelvin G. K. Goh, Lahiru Katupitiya, Dean Gosling, Michelle N. Chamoun, Asha Kakkanat, Debasish Chattopadhyay, Michael Crowley, David K. Crossman, Mark A. Schembri, Glen C. Ulett

**Affiliations:** a School of Medical Sciences and Menzies Health Institute Queensland, Griffith University, Parklands, Australia; b School of Medicine, Division of Infectious Diseases, University of Alabama at Birmingham, Birmingham, Alabama, USA; c Heflin Center for Genomic Sciences, University of Alabama at Birmingham, Birmingham, Alabama, USA; d School of Chemistry and Molecular Biosciences, University of Queensland, Brisbane, Australia; University of Kentucky

**Keywords:** flagella, urinary tract infection, uropathogenic *Escherichia coli*

## Abstract

Urinary tract infection (UTI) caused by uropathogenic Escherichia coli (UPEC) engages interleukin-10 (IL-10) as an early innate immune response to regulate inflammation and promote the control of bladder infection. However, the mechanism of engagement of innate immunity by UPEC that leads to elicitation of IL-10 in the bladder is unknown. Here, we identify the major UPEC flagellar filament, FliC, as a key bacterial component sensed by the bladder innate immune system responsible for the induction of IL-10 synthesis. IL-10 responses of human as well as mouse bladder epithelial cell-monocyte cocultures were triggered by flagella of three major UPEC representative strains, CFT073, UTI89, and EC958. FliC purified to homogeneity induced IL-10 *in vitro* and *in vivo* as well as other functionally related cytokines, including IL-6. The genome-wide innate immunological context of FliC-induced IL-10 in the bladder was defined using RNA sequencing that revealed a network of transcriptional and antibacterial defenses comprising 1,400 genes that were induced by FliC. Of the FliC-responsive bladder transcriptome, altered expression of *il10* and 808 additional genes were dependent on Toll-like receptor 5 (TLR5), according to analysis of TLR5-deficient mice. Examination of the potential of FliC and associated innate immune signature in the bladder to boost host defense, based on prophylactic or therapeutic administration to mice, revealed significant benefits for the control of UPEC. We conclude that detection of FliC through TLR5 triggers rapid IL-10 synthesis in the bladder, and FliC represents a potential immune modulator that might offer benefit for the treatment or prevention of UPEC UTI.

**IMPORTANCE** Interleukin-10 is part of the immune response to urinary tract infection (UTI) due to E. coli, and it is important in the early control of infection in the bladder. Defining the mechanism of engagement of the immune system by the bacteria that enables the protective IL-10 response is critical to exploring how we might exploit this mechanism for new infection control strategies. In this study, we reveal part of the bacterial flagellar apparatus (FliC) is an important component that is sensed by and responsible for induction of IL-10 in the response to UPEC. We show this response occurs in a TLR5-dependent manner. Using infection prevention and control trials in mice infected with E. coli, this study also provides evidence that purified FliC might be of value in novel approaches for the treatment of UTI or in preventing infection by exploiting the FliC-triggered bladder transcriptome.

## Abstract (precis)

Interleukin-10 is part of the immune response to urinary tract infection (UTI) due to E. coli, and it is important in the early control of infection in the bladder. Defining the mechanism of engagement of the immune system by the bacteria that enables the protective IL-10 response is critical to exploring how we might exploit this mechanism for new infection control strategies. In this study, we reveal part of the bacterial flagellar apparatus (FliC) is an important component that is sensed by and responsible for induction of IL-10 in the response to UPEC. We show this response occurs in a TLR5-dependent manner. Using infection prevention and control trials in mice infected with E. coli, this study also provides evidence that purified FliC might be of value in novel approaches for the treatment of UTI or in preventing infection by exploiting the FliC-triggered bladder transcriptome.

## INTRODUCTION

Urinary tract infections (UTI) are common illnesses, predominantly affecting women and causing more than ten million ambulatory visits per year in the United States alone (1). Expenditures aimed at the management of UTI account for approximately $3.5 billion in medical costs annually (2). Up to 80% of acute UTI cases are caused by uropathogenic Escherichia coli (UPEC) (3). Studies have shown key roles for virulence factors of UPEC, such as flagella, autotransporters, capsule, fimbriae, toxins, lipopolysaccharide (LPS), and siderophores, in UTI disease pathogenesis (4–7).

The innate immune signature of acute UPEC UTI is reviewed elsewhere (7); it encompasses various cytokines, including interleukin-10 (IL-10), that is upregulated in the bladder within a few hours of experimental infection in mice (8). IL-10 is secreted in urine of adults who exhibit symptomatic UTI (8, 9) and is induced in several *in vitro* models of UTI, including in monocytes and mast cells (10, 11) and bladder epithelial cell-monocyte cocultures (10), which are used to model host-pathogen interactions (12). IL-10 plays pleiotropic roles in defense against infection depending on the illness and the causal pathogen. Frequently, IL-10 facilitates immune suppression to moderate inflammatory mechanisms that can damage the host (13–16). The contribution of IL-10 to resolution of infection reflects its tightly controlled expression, which can be a key factor in determining disease outcome (17–19). Functionally, an absence of IL-10 in mice exacerbates the host’s ability to control bacterial colonization during the innate phase of infection in the bladder (8). Reflecting its central regulatory role in many diseases and its ability to reduce tissue damage and protect tissue integrity, IL-10 is the subject of clinical trials for inflammatory diseases; however, its manipulation for benefit in a therapeutic setting remains experimental (20).

One facet in understanding the role of IL-10 in infectious disease is elucidation of microbial products that elicit production of this key regulator of innate immune responses. Bacterial virulence factors shown to induce the production of IL-10 in experimental disease models include M protein of *Streptococcus* (21), peptidoglycan-embedded lipopeptides and cell wall glycopolymers of *Staphylococcus* (22), and flagella of *Salmonella* (23) and *Yersinia* (24). For some other pathogens that trigger the production of IL-10, including *Helicobacter* and *Chlamydia*, links between virulence factors and IL-10 elicitation remain elusive. The nature of the host response encompassing IL-10 can also depend on the genus or species of origin from which the pathogen virulence factor is derived (25–32).

Flagella of UPEC contribute to the pathogenesis of UTI in several ways, including through motility that is associated with bacterial ascension from the bladder to the kidneys, leading to the development of pyelonephritis (33, 34). Expression of flagella by UPEC has also been associated with enhanced urinary tract colonization, invasion of host cells (35, 36), survival inside macrophages (37), and biofilm formation (38, 39). The flagellar filament is synthesized as a polymerized product of >20,000 protein monomers, termed flagellin or FliC (usually encoded by *fliC*), as reviewed elsewhere (40). In mammals, flagella are characteristically sensed through Toll-like receptor 5 (TLR5), which recognizes FliC monomers but not flagellar filaments (41–45). FliC can also be detected by NLR family apoptosis inhibitory protein 5 (NAIP5) and Ipaf within the intracellular environment (46, 47). Initial observations suggested that TLR11 senses flagellin (48–51); however, it has since been established that binding of flagellin to TLR11 does not occur, and the responses of wild-type and TLR11-deficient mice to flagellin are similar (52). A detailed understanding of how FliC from UPEC engages innate immunity in the bladder during UTI is lacking (34, 53–55), and the potential contribution of FliC to rapid IL-10 induction in the bladder during UTI is unknown. In this study, we examined the role of FliC in the bladder innate immune response to UPEC, with a focus on early IL-10 induction and the role of TLR5 in the FliC-driven bladder defense response.

## RESULTS

### Effect of flagellar expression on UPEC-induced IL-10 in uroepithelial cell monocyte cocultures.

**FIG 1:**
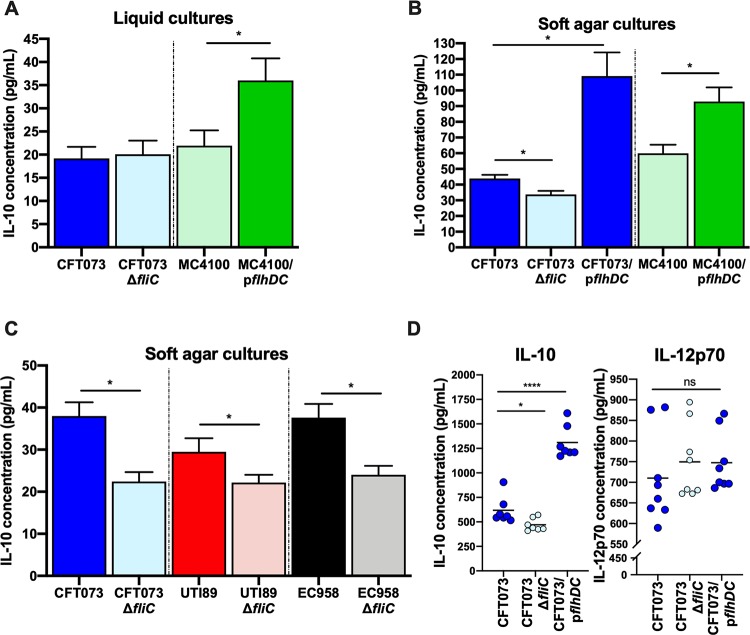
IL-10 production in uroepithelial cell monocyte cocultures challenged with UPEC CFT073 and other E. coli strains with altered flagellar expression. (A) Human 5637-U937 cocultures exposed to liquid-grown CFT073 and *fliC*-deficient CFT073 or MC4100 with or without p*flhDC* for hyperflagellation. Significance was determined by *t* test for MC4100 versus MC4100/p*flhDC* (*, *P* = 0.02). (B) Human cell cocultures exposed to soft-agar-grown CFT073 or *fliC*-deficient and p*flhDC* derivatives and MC4100 strains. Significance was determined by ANOVA for CFT073 strains and *t* test for MC4100 strains (*, *P* = 0.02). (C) Responses of human 5637-U937 cocultures to CFT073, UTI89, and EC958 (soft agar grown) and their *fliC*-deficient mutants. (D) Responses of IL-10 and the functionally opposed cytokine IL-12p70 in cocultures exposed to CFT073, CFT073Δ*fliC*, and CFT073/p*flhDC* strains according to multiplex analysis. Significance was determined by ANOVA with Tukey’s *post hoc* analysis (*, *P* < 0.05). Additional responses of other cytokines and chemokines are illustrated in Fig. S1.

In initial experiments testing the effect of differential UPEC flagellar expression on IL-10 induction, we used liquid-grown wild-type (WT) and *fliC*-deficient CFT073 and E. coli MC4100 (deficient for flagella due to a frameshift mutation in the *flhD* master regulator [56]) and MC4100/p*flhDC* (p*flhDC* was used to confer a hyperflagellated state) (Table 1). Uroepithelial cell-monocyte cocultures exhibited a 7-fold increase in IL-10 at 5 h after infection with MC4100/p*flhDC* and a 4-fold increase for other infections (on average) versus noninfected controls (Fig. 1A). The level of IL-10 induced by MC4100/p*flhDC* compared to that of MC4100 WT was statistically significant (*P* = 0.02); there was no difference between CFT073 WT and CFT073Δ*fliC* strains under these conditions. We next tested bacteria grown on soft agar, which induces swarming associated with increased flagellin expression. In these assays, CFT073 WT induced significantly more IL-10 than the CFT073Δ*fliC* strain but less IL-10 than CFT073/p*flhDC* (Fig. 1B). Similar IL-10 responses occurred in cultures exposed to MC4100 WT and MC4100/p*flhDC*. Experiments comparing the responses of human cell cocultures to UPEC UTI89 and EC958 and their respective *fliC*-deficient derivatives showed equivalent trends in which higher levels of IL-10 were induced by UPEC expressing flagella than *fliC*-deficient mutants (Fig. 1C).

**TABLE 1:** Bacterial strains and plasmids used in this study

Strain or plasmid	Characteristic(s)	Reference or source
E. coli strains		
DH5α	Cloning strain; d*lacZ*ΔM15 Δ(*lacZYA*-*argF*)*U169 recA1 endA1 hsdR17*(r_K_^−^ m_K_^+^) *supE44 thi-1 gyrA96 relA1*	Bethesda Research Laboratories
MC4100	E. coli K-12 strain, OR:H48	111
MC4100/p*flhDC*	MC4100 containing p*flhDC*; Kn^r^	112
CFT073	Reference UPEC strain, O6:K2:H1 (ATCC 700928)	101
UTI89	Reference UPEC strain, O18:K1:H7	102
EC958	Reference ST131 UPEC strain, O25b:K100:H4	103
GU2139	CFT073/p*flhDC*; Kn^r^	57
GU2639	CFT073Δ*fliC*; Kn^r^	57
GU2671	UTI89Δ*fliC*; Kn^r^	This study
EC958Δ*fliC*	EC958Δ*fliC*; Cm^r^	68
CFT073*Δ4*	CFT073 with combined deletions Δ*fim*, Δ*foc*, Δ*pap1*, and Δ*pap2*	113
GU2647	CFT073Δ*4*/p*flhDC* (p*flhDC*); Kn^r^	57
GU2642	CFT073Δ*4* Δ*fliC*; *fliC*^−^ derivative of CFT073Δ*4*	57
GU2648	GU2642/p*flhDC*; Kn^r^ (for carrier control)	57
Plasmids		
p*flhDC*	*flhDC* operon from *Serratia* in pVLT33; Cm^r^	112
pKD4	Template plasmid for *kan* gene amplification	104
pKD46	λ-Red recombinase expression plasmid	104
pCP20	FLP synthesis under thermal control	104

10.1128/mSphere.00545-19.1 FIG S1 Multiplex analysis of cytokine production in uroepithelial cell monocyte cocultures challenged with UPEC CFT073 and other E. coli strains with altered flagellar expression. Responses in human cell cocultures exposed to medium control (Ctrl), CFT073 (C-Wt), CFT073/p*flhDC* (C-Wt-pMG), and CFT073Δ*fliC* (C-FliC-) for 5 h at an MOI of 10. Significant responses include those of IFN-γ, TNF-α, GM-CSF, IL-17, MCP-1, IP-10, eotaxin, and MIP-1α/β, among many others. Significance was determined by ANOVA with Tukey’s post hoc analysis: *, *P* < 0.05, C-Wt-pMG versus C-Wt or C-FliC-; #, *P* < 0.05, C-Wt versus C-FliC-. Download FIG S1, TIF file, 0.5 MB. Copyright © 2019 Acharya et al. 2019 Acharya et al. This content is distributed under the terms of the Creative Commons Attribution 4.0 International license.

Measurement of IL-10 induction in response to UPEC CFT073 and derivatives was then undertaken using a multiplex assay to explore functionally opposed (e.g., IL-12p70, IL-2, and tumor necrosis factor alpha [TNF-α]) and related cytokines (e.g., IL-4 and IL-6) and thereby gain a broader picture of the immunological context of IL-10 induction (14). In contrast to the induction of IL-10 observed in the response to hyperflagellated UPEC CFT073/p*flhDC* compared to WT (and significantly lower levels than the CFT073Δ*fliC* strain), there were no changes in levels of IL-12p70 (Fig. 1D); however, statistically significant changes in several other cytokines, including IL-1α, -1β, -2, -4, -6, and TNF-α, and multiple chemokines (e.g., granulocyte colony-stimulating factor [G-CSF]) were detected (see Fig. S1 in the supplemental material). Hyperflagellation in *flhDC*-complemented UPEC strains and an absence of flagellar expression in *fliC*-deficient strains was confirmed using immunoblots for FliC (Fig. S2A), as previously described (57); in addition, motility assays showed phenotypes consistent with hyperflagellation in *flhDC*-complemented UPEC strains (Fig. S2B). Taken together, these data show that flagellar expression in CFT073 and other UPEC strains, including UTI89 and EC958, induces the production of IL-10 in uroepithelial cell monocyte cocultures as well as significant induction of several other functionally opposed and related cytokines.

10.1128/mSphere.00545-19.2 FIG S2 Hyperflagellation in *flhDC*-complemented UPEC CFT073. Immunoblot for FliC using whole lysates of UPEC prepared from overnight liquid cultures (A) and results of motility assays (B), demonstrating phenotypes consistent with hyperflagellation in UPEC CFT073 strains carrying p*flhDC* (WT and CFT073Δ*4* shown) and an absence of FliC in CFT073Δ*fliC* and CFT073Δ*4*Δ*fliC* strains. Motility assay data are shown as the mean diameter (mm) ± SEM of growth on soft agar for at least 3 independent experiments. Download FIG S2, PDF file, 1.5 MB. Copyright © 2019 Acharya et al. 2019 Acharya et al. This content is distributed under the terms of the Creative Commons Attribution 4.0 International license.

### IL-10 responses of cell cultures to enriched flagella and purified FliC.

**FIG 2:**
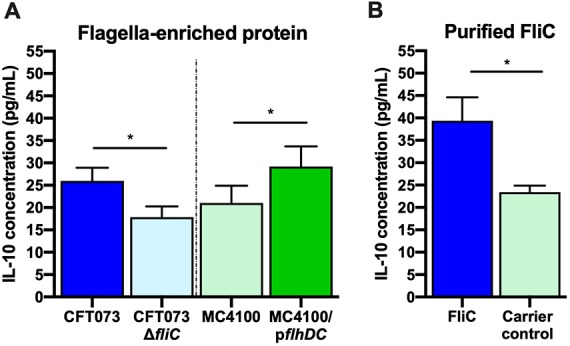
IL-10 production in human cells *in vitro* after stimulation with flagella and purified FliC from UPEC CFT073. (A) Human uroepithelial cell monocyte cocultures stimulated (5 h) with flagellum-enriched protein (1 μg) from CFT073 and CFT073Δ*fliC* strains or MC4100 with or without p*flhDC*. Significance was determined by *t* test for CFT073 strains (*, *P* < 0.05). (B) Monocytes stimulated (5 h) with purified FliC (1 μg) from CFT073Δ*4* strain or carrier control (generated from CFT073Δ*4*Δ*fliC*). Significance was determined by *t* tests (*, *P* < 0.05).

Experiments examining the responses of uroepithelial cell monocyte cocultures to flagellum-enriched protein from CFT073 (isolated by shearing and ultracentrifugation) showed significant IL-10 responses to flagella from CFT073 WT (versus the CFT073Δ*fliC* mutant) and MC4100/p*flhDC* (versus MC4100 WT) (Fig. 2A). Subsequently, we measured IL-10 levels in response to FliC purified to homogeneity from CFT073Δ*4*/p*flhDC* using fast protein liquid chromatography (FPLC), because flagellum-enriched preparations contain trace amounts of other outer membrane proteins that could contribute to IL-10 induction (57). Pure FliC triggered significantly more IL-10 than the carrier control that was generated from the CFT073Δ*4* Δ*fliC* strain (Fig. 2B). Similar responses were observed for mouse macrophages but did not reach statistical significance due to higher basal levels of IL-10 detected in these experiments (data not shown). Taken together, these findings show that pure FliC stimulates significant IL-10 synthesis in human uroepithelial cell monocyte cocultures.

### IL-10 and related responses of the mouse bladder to FliC.

**FIG 3:**
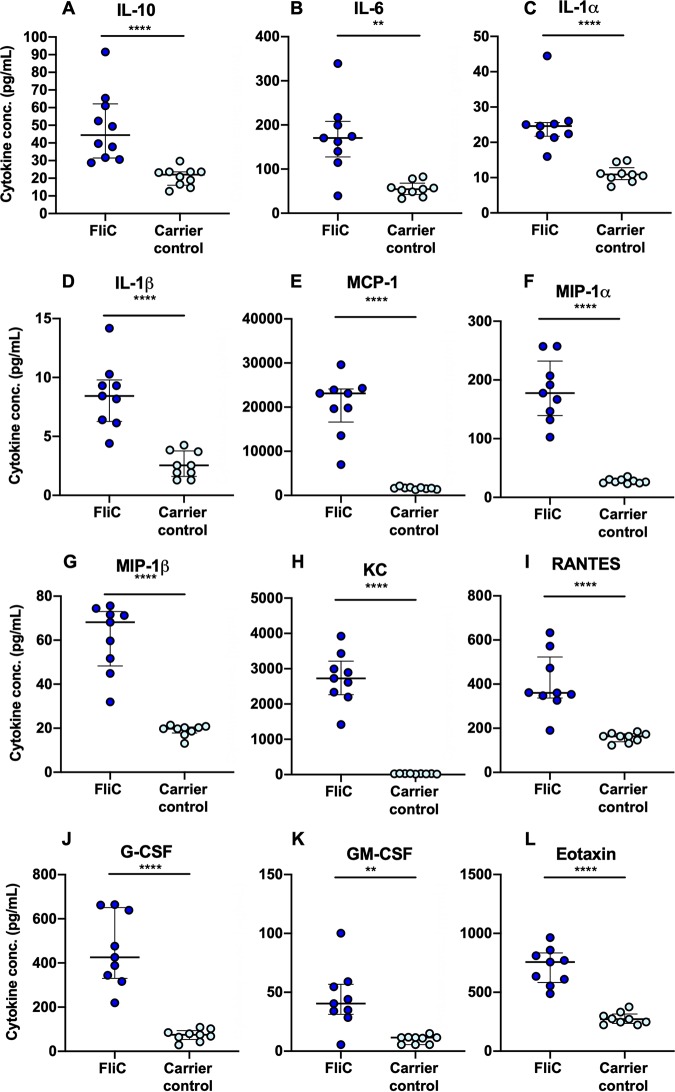
Bladder IL-10 and other cytokine responses in mice treated with purified FliC from UPEC CFT073Δ*4* strain. Multiplex analysis of IL-10 and other cytokines in bladder homogenates at 2 h following transurethral delivery of 30 μg FliC or carrier control. Significance was determined by *t* test for FliC versus the control (*, *P* < 0.05; ****, *P* < 0.0001). All cytokines that exhibited significantly altered expression are shown, with additional multiplex data (for nonsignificant factors) provided in Fig. S3. Data shown represent at least 2 independent experiments with separate groups of mice (*n* = 9 [at least] per group).

We next analyzed the bladder response in mice that received either 30 μg of pure FliC from CFT073Δ*4*/p*flhDC* or the equivalent volume of carrier control generated from the CFT073Δ*4* Δ*fliC*/p*flhDC* strain. Transurethral delivery of FliC triggered significant production of IL-10 in the bladders of mice at 2 h postinoculation compared to that of control mice according to multiplex assay (Fig. 3A). Levels of IL-6, often associated with IL-10-regulated responses, were also elevated (Fig. 3B), as were levels of IL-1α, IL-1β, and several chemokines, including monocyte chemoattractant protein 1 (MCP-1/CCL2), macrophage inflammatory protein 1α (MIP-1α/CCL3) and -β (MIP-1β/CCL4), and RANTES (CCL5) (Fig. 3). However, there were no significant changes in levels of IL-12p40, IL-12p70, TNF-α, IL-2, IL-4, IL-5, or IL-13 (Fig. S3). Data generated using enzyme-linked immunosorbent assay (ELISA) for IL-10 were consistent with elevated levels of IL-10, as detected by multiplex assay (Fig. S4). Thus, UPEC FliC causes rapid induction of IL-10 in the bladder with concurrent early responses for IL-6, IL-1, and multiple chemokines.

10.1128/mSphere.00545-19.3 FIG S3 Multiplex analysis of cytokine levels in bladders of mice treated with purified FliC from UPEC CFT073Δ*4*. Responses are at 2 h following transurethral delivery of 30 μg FliC (in 50 μl carrier) or an equivalent volume of carrier control. Significant responses include those of IL-1α, IL-1β, and multiple chemokines, such as monocyte chemoattractant protein-1 (MCP-1/CCL2), macrophage inflammatory protein 1α (MIP-1α/CCL3) and -β (MIP-1β/CCL4), and RANTES (CCL5). Significance was determined by ANOVA with Tukey’s post hoc analysis (*, *P* < 0.05). Download FIG S3, TIF file, 0.6 MB. Copyright © 2019 Acharya et al. 2019 Acharya et al. This content is distributed under the terms of the Creative Commons Attribution 4.0 International license.

10.1128/mSphere.00545-19.4 FIG S4 ELISA of IL-10 levels in bladders of mice treated with purified FliC from UPEC CFT073Δ*4*. Responses are at 2 h following transurethral delivery of 30 μg FliC (in 50 μl carrier) or an equivalent volume of carrier control. Data shown represent 2 independent experiments with separate groups of mice (*n* = 10 per group). The groups were compared using *t* test (*, *P* < 0.05). Download FIG S4, TIF file, 0.1 MB. Copyright © 2019 Acharya et al. 2019 Acharya et al. This content is distributed under the terms of the Creative Commons Attribution 4.0 International license.

### The FliC-responsive bladder transcriptome and dependency on TLR5.

**FIG 4:**
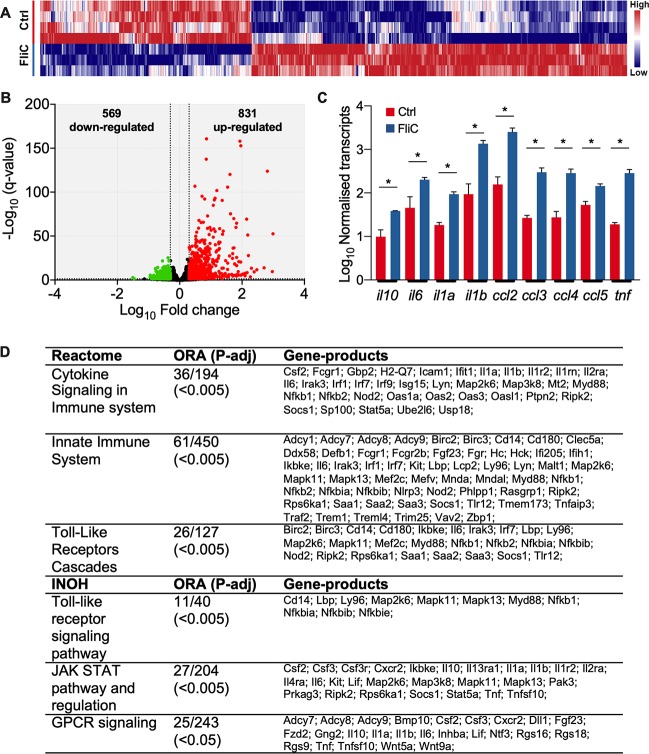
Bladder transcriptome in WT mice in response to pure FliC from UPEC CFT073Δ*4*. (A) Heat map of transcriptional changes in mouse bladder in response to 30 μg pure FliC (in 50 μl carrier) or equivalent volume of carrier control (Ctrl) (2-h exposure). (B) Volcano plot of the total number and the breadth of fold change of transcriptional response of genes exhibiting significantly altered expression (fold change of ≥±2.0, *q* value of <0.05) in the bladder response to pure FliC. (C) Normalized transcript abundances for *il10* and several other genes encoding cytokines in the FliC-treated and carrier control groups (bars represent the means ± SEM; an asterisk denotes a fold change of ≥±2.0 and *q* value of <0.05. (D) Top canonical biological pathways, according to Reactome (upper) and Integrating Network Objects with Hierarchies (INOH) analysis (lower).

We next defined a more complete picture of the innate immune response of mouse bladder to FliC using RNA sequencing to comprehensively map the transcriptional responses that initiated with early IL-10 induction. Bladders of WT mice exposed to FliC or carrier control exhibited distinct global transcriptional signatures (Fig. 4A) that encompassed 1,400 significant gene responses, represented by 831 upregulated and 569 downregulated genes (Fig. 4B); significance criteria included a fold change of ≥±2.0 and *q* value of <0.05, as described in Materials and Methods. Upregulated genes of particular interest in the context of IL-10 and innate immune activation included *il10* (3.3-fold), *il6* (3.2-fold), *il1a* (5.1-fold), *il1b* (10.9-fold), *ccl2* (14.0-fold), *ccl3* (11.5-fold), *ccl4* (9.6-fold), *ccl5* (2.6-fold), and *tnf* (15.5-fold); the responses of these genes are illustrated as absolute transcript abundance for control and FliC groups in Fig. 4C. The complete list of significant gene responses is listed in Data Set S1. The *tlr5* gene was significantly downregulated (2.3-fold). Notably, many transcriptional responses detected by RNA sequencing exhibited consistency with parallel translational activities detected in the bladder, including those for IL-10, -6, and -1 and chemokines, according to the multiplex protein assays (Fig. 3 and Fig. S4).

10.1128/mSphere.00545-19.8 DATA SET S1 Bladder transcriptome in WT mice in response to pure FliC from UPEC CFT073Δ*4*. Spreadsheets (left to right) represent the comparison of FliC-treated WT mice to carrier control-treated WT mice and list transcriptional response of genes exhibiting significantly altered expression in the bladder in response to FliC and significant biological pathways activated according to innateDB analysis (listing Reactome, INOH, and KEGG outputs). The analysis parameters are described in the right-most tab. Download Data Set S1, XLSX file, 10.7 MB. Copyright © 2019 Acharya et al. 2019 Acharya et al. This content is distributed under the terms of the Creative Commons Attribution 4.0 International license.

The top five canonical pathways (generated by Reactome analysis within innateDB [58] and ranked according to significance from overrepresentation analysis [ORA]) are summarized in Fig. 4D. These data highlight the extensive activation of networks related to cytokine signaling in the innate immune system and TLR cascades activated as a result of FliC treatment (complete list is in Data Set S1). Integrating Network Objects with Hierarchies (INOH) analysis identified similar strongly activated biological processes in FliC-treated WT mice, including TLR signaling, JAK STAT pathway activity, and GPCR signaling (Fig. 4D and Data Set S1). Taken together, these data illustrate an overall FliC-responsive bladder transcriptome that is characterized by extensive cytokine and TLR signaling and innate immune regulatory processes that are collectively engaged with early *il10* induction.

**FIG 5:**
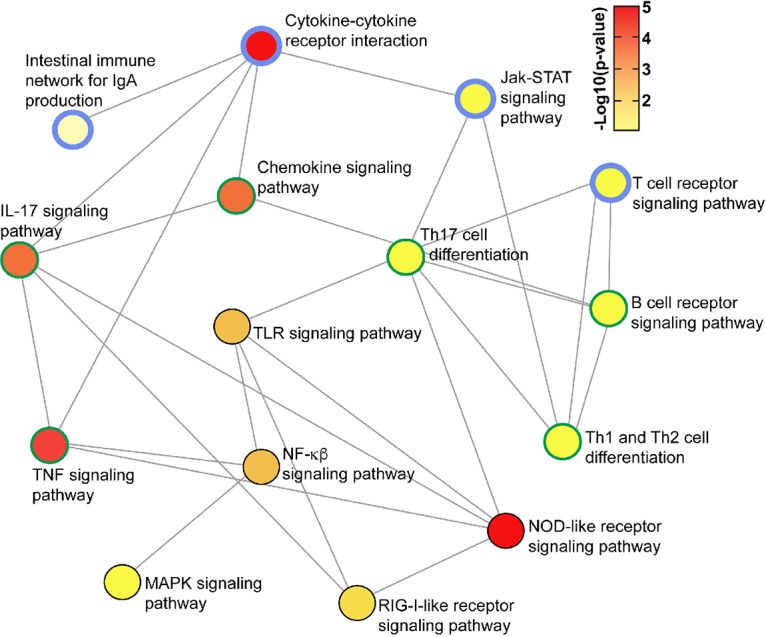
Topology network of interactive elements of the TLR5-dependent, FliC-responsive bladder transcriptome. The network highlights key nodes that include *il10* (blue edge) at the top of the network and the nodes that are directly (green edge) and indirectly (black edge) associated with *il10*-containing nodes. The network incorporates significant elements of cytokine-cytokine receptor interactions, IL-17 and chemokine signaling, lymphocyte signaling and differentiation, and underlying signaling pathways, such as those for NF-κβ and MAPK. Images were derived using Network Analyst (59) and based on KEGG ontologies, with colors related to the significance of pathway activation.

**FIG 6:**
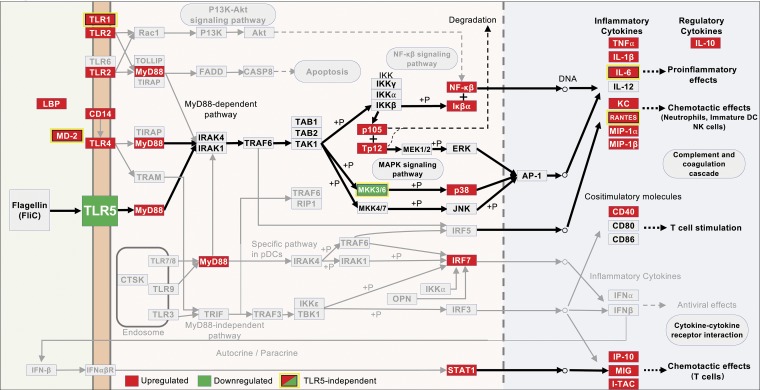
Cellular context of TLR5 engagement by UPEC FliC in the bladder leading to early IL-10 induction. Gene transcriptional responses analyzed using innateDB and overlaid on KEGG pathway 4620 Toll-like receptor signaling. Color key: green, downregulated; red, upregulated; yellow box, TLR5-dependent; other diagram components are per KEGG definitions. The illustration highlights possible signaling transduction mechanisms (center) that are engaged by FliC, leading to rapid IL-10 synthesis in the bladder. IL-10 does not form part of the canonical KEGG pathway 4620 but is included as a notional product of TLR5 engagement based on the findings of this study.

Comparative analysis of TLR5-deficient mice enabled delineation of the bladder responses of WT mice that are contingent on TLR5; this identified a total of 809 genes (652 upregulated, 157 downregulated), including *il10*, that depend on TLR5 for their activation or repression in response to FliC; these are illustrated according to topology analysis of key nodes in Fig. 5 (59). Heat maps and a volcano plot representing the FliC-responsive bladder transcriptome of WT and TLR5-deficient mice are shown in Fig. S5. The complete list of bladder transcriptional responses and biological pathways activated in response to FliC in a TLR5-dependent manner (i.e., WT versus TLR5-deficient mice) is provided in Data Set S2 (also provides a complete list of TLR5-independent responses). Unexpectedly, this analysis also revealed 591 genes that exhibited significantly altered expression in response to FliC independent of TLR5; this comprised 591 genes (179 upregulated and 412 downregulated genes; i.e., responses exclusive to FliC-treated WT versus carrier-treated WT mice and absent from a comparison of FliC-treated WT versus FliC-treated TLR5-deficient mice). A summary of the top 30 gene responses triggered by FliC via TLR5-dependent and -independent mechanisms is provided in Table 2. A visual summary of the responses in the form of a Venn diagram is provided in Fig. S6. The cellular context of TLR5-dependent and -independent responses identified genes with altered expression in the significantly activated TLR signal transduction pathway, as defined by innateDB and KEGG (Fig. 6). Taken together, these data establish that *il10* transcriptional activation is part of a rapid bladder defense strategy in response to FliC, which occurs in a TLR5-dependent manner; additionally, *il10* is part of a broader response that is initiated concurrently with an assembly of other TLR5-dependent, as well as TLR5-independent, transcriptional responses.

**TABLE 2:** Top 30 genes in the bladder transcriptional response to FliC that are altered in expression via TLR5-dependent and -independent mechanisms

Gene	Fold change^*a*^	*P* value	Annotation
Upregulated TLR5-dependent response			
* ccl20*	980.3	4.94E−56	Chemokine (C-C motif) ligand 20
* ngp*	934.1	5.55E−12	Neutrophilic granule protein
* slc6a14*	647.2	5.13E−128	Solute carrier family 6, member 14
* sprr2e*	510	1.85E−16	Small proline-rich protein 2E
* adgrf1*	298	3.19E−14	Adhesion G protein-coupled receptor F1
* saa1*	245	1.33E−11	Serum amyloid A1
* sprr2d*	199.8	5.15E−16	Small proline-rich protein 2D
* saa2*	185.1	1.70E−05	Serum amyloid A1
* ltf*	159.9	7.92E−31	Lactotransferrin
* abcc8*	152	1.45E−54	ATP-binding cassette, subfamily C member 8
* olfm4*	145.6	1.06E−10	Olfactomedin 4
* gm16685*	140.2	6.06E−73	Predicted gene, 16685
* gm5483*	123.7	1.11E−07	Predicted gene, 5483
* sprr2h*	123.4	5.96E−10	Small proline-rich protein 2H
* slc26a4*	104.4	8.14E−09	Solute carrier family 26, member 4
Downregulated TLR5-dependent response			
* fam131b*	−4.7	2.84E−03	Family with sequence similarity 131, member B
* oprk1*	−4.8	3.69E−04	Opioid receptor, kappa 1
* pnmal2*	−4.9	1.38E−05	PNMA-like 2
* bach2*	−5.1	5.86E−17	BTB and CNC homology, basic leucine zipper transcription factor 2
* gm4869*	−5.3	1.05E−08	Predicted gene, 4869
* slc16a14*	−5.3	2.76E−12	Solute carrier family 16 (monocarboxylic acid transporters), member 14
* gas1*	−5.4	4.31E−05	Growth arrest specific 1
* rbbp8nl*	−5.4	2.87E−06	RBBP8 N-terminal like
* oprd1*	−6	1.89E−15	Opioid receptor, delta 1
* alkal1*	−6.1	1.88E−03	ALK and LTK ligand 1
* evx2*	−6.8	1.71E−07	Even-skipped homeobox 2
* fam47e*	−6.9	1.42E−03	Family with sequence similarity 47, member E
* gm15513*	−7.4	1.83E−03	Predicted gene, 15513
* foxn1*	−7.6	1.69E−05	Forkhead box N1
* gm37711*	−8.8	9.75E−08	Predicted gene, 37711
Upregulated TLR5-independent response			
* mrgpra2b*	124.57	1.02E−06	MAS-related GPR, member A2B
* mir351*	27.64	2.83E−06	MicroRNA 351
* igkv12-89*	22.77	6.51E−03	Immunoglobulin kappa chain variable 12-89
* gbp6*	12.18	2.48E−21	Guanylate binding protein 6
* gm9378*	10.82	8.14E−05	Predicted gene 9378
* gm24245*	9.86	3.22E−03	Predicted gene 24245
* fam26f*	9.05	5.11E−20	Family with sequence similarity 26, member F
* gm43305*	7.47	6.25E−03	Predicted gene 43305
* c030013C21Rik*	6.46	1.47E−07	RIKEN cDNA C030013C21 gene
* slpi*	6.12	2.06E−21	Secretory leukocyte peptidase inhibitor
* clca3b*	6.08	7.96E−03	Chloride channel accessory 3B
* ang4*	5.86	2.09E−03	Angiogenin, ribonuclease A family, member 4
* rem2*	5.51	3.73E−03	rad- and gem-related GTP binding protein 2
* ldoc1*	5.51	7.72E−05	Regulator of NFKB signaling
* gm8818*	4.66	9.33E−04	Predicted pseudogene 8818
Downregulated TLR5-independent response			
* gm34583*	−6.32	4.17E−05	Predicted gene 34583
* pcdhb2*	−6.41	1.96E−03	Protocadherin beta 2
* 5830418P13Rik*	−6.45	1.90E−03	RIKEN cDNA 5830418P13 gene
* slc6a11*	−6.45	5.46E−03	Solute carrier family 6 (neurotransmitter transporter, GABA), member 11
* gm43480*	−6.49	2.26E−03	Predicted gene 43480
* gsdmc*	−6.55	1.64E−05	Gasdermin C
* slitrk3*	−7.03	6.16E−04	SLIT and NTRK-like family, member 3
* ucp3*	−7.24	2.00E−05	Uncoupling protein 3 (mitochondrial, proton carrier)
* gbp2b*	−7.57	1.13E−02	Guanylate binding protein 2b
* lrtm1*	−8.08	5.20E−03	Leucine-rich repeats and transmembrane domain 1
* ascl1*	−8.15	3.06E−04	Achaete-scute family bhlh transcription factor 1
* htr4*	−8.88	3.22E−05	5 Hydroxytryptamine (serotonin) receptor 4
* gm35507*	−29.13	3.14E−03	Predicted gene 35507
* otop1*	−29.48	7.62E−03	Otopetrin 1
* mmd2*	−31.89	2.08E−04	Monocyte to macrophage differentiation-associated 2

a Fold change refers to gene expression in the bladders of WT mice treated with FliC relative to carrier control.

10.1128/mSphere.00545-19.5 FIG S5 Comparative bladder transcriptome of WT versus B6.129S1-Tlr5^tm1Flv/^J mice stimulated with pure FliC. (A) Heat map of transcriptional changes in WT mouse bladder in response to 30 μg pure FliC (in 50 μl carrier) or B6.129S1-Tlr5^tm1Flv^/J mouse bladder in response to 30 μg pure FliC (in 50 μl carrier; 2-h exposure). (B) Volcano plot of the total number and the breadth of fold change of transcriptional response of genes exhibiting significantly altered expression in the bladder response to FliC. (C) Normalized transcript abundances for *il-10* and several other cytokine genes in the FliC-treated WT and FliC-treated B6.129S1-Tlr5^tm1Flv^/J mouse groups (bars represent the means ±SEM; an asterisk denotes a fold change of ≥±2.0 and *q* value of <0.05. Download FIG S5, PDF file, 1.3 MB. Copyright © 2019 Acharya et al. 2019 Acharya et al. This content is distributed under the terms of the Creative Commons Attribution 4.0 International license.

10.1128/mSphere.00545-19.6 FIG S6 Summary of TLR5-dependent and -independent elements of the FliC-responsive bladder transcriptome. (A and B) Venn diagrams showing the number of upregulated (A) and downregulated (B) genes that are shared between the comparisons in this study. Of the 831 up- and 569 downregulated genes responding to FliC in the WT mice versus the control, 809 of these (652 up- and 157 downregulated) were also differentially regulated in the WT mice with FliC compared to that in the *tlr5*-deficient mice with FliC. Thus, inactivation of TLR5 abrogated their expression, indicating that these responses are dependent on TLR5 (maroon; FliC responses dependent on TLR5). There were an additional 591 genes (179 up- and 412 downregulated) that changed only in response to FliC (i.e., no difference between WT or *tlr5*-deficient mice following FliC stimulation) and were independent of TLR5 signaling (blue; effect of FliC, independent of TLR5) and a further 1,040 gene changes that were attributed to the *tlr5* mutation, independent of FliC stimulation (red; effect of TLR5, independent of FliC). The diagram was constructed using Venny (https://bioinfogp.cnb.csic.es/tools/venny/index.html). The complete gene lists used to generate the diagram are provided in Data Set S2. Download FIG S6, PDF file, 0.4 MB. Copyright © 2019 Acharya et al. 2019 Acharya et al. This content is distributed under the terms of the Creative Commons Attribution 4.0 International license.

10.1128/mSphere.00545-19.9 DATA SET S2 Elements of the FliC-responsive bladder transcriptome in WT mice that are engaged via TLR5-dependent and -independent mechanisms. The spreadsheets (left to right) represent the comparison of FliC-treated WT mice to FliC-treated B6.129S1-Tlr5^tm1Flv^/J mice and list transcriptional response of genes exhibiting significantly altered expression in the bladder response to FliC and significant biological pathways activated according to innateDB analysis (listing Reactome, INOH, KEGG outputs, and innateDB parameters). The spreadsheets in the right-most tabs (S3 and S4) show a compiled TLR5-dependent response (i.e., shared between gene lists of Data Sets S1 and S2 for comparison of FliC-treated WT mice to FliC-treated B6.129S1-Tlr5^tm1Flv^/J mice) and TLR5-independent responses (i.e., unique to the FliC versus Ctrl WT comparison). Partner Venn diagrams to visually summarize these lists are provided in Fig. S6. Download Data Set S2, XLSX file, 0.4 MB. Copyright © 2019 Acharya et al. 2019 Acharya et al. This content is distributed under the terms of the Creative Commons Attribution 4.0 International license.

### Controlling UPEC UTI through FliC-mediated innate immunity.

**FIG 7:**
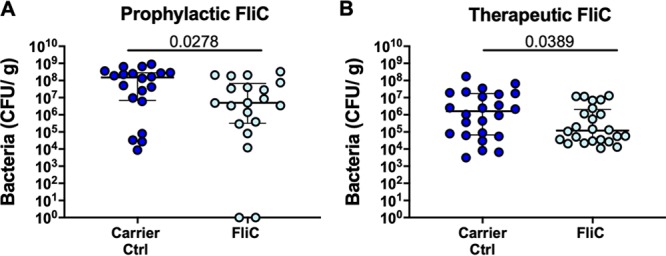
Control of UPEC UTI by FliC treatment. (A) Prophylactic FliC was administered to the bladders of mice 2 h prior to infectious challenge with UPEC. (B) Therapeutic FliC was administered to the bladders of mice 24 h after infectious challenge with UPEC. Bacterial loads were determined at 24 h (A) and 48 h (B) after infectious challenge. Both prophylactic and therapeutic FliC treatment significantly reduced the numbers of UPEC recovered from the bladders of mice treated with FliC compared to control mice that received carrier alone. Data for urine and kidneys are provided in Fig. S7. Data shown represent pooled data from 2 to 3 independent experiments, each comprising 8 to 10 mice per group (total *n* = 20 to 30 per group). *, *P* < 0.05 by Mann-Whitney U test (data did not satisfy Gaussian distribution or normality tests).

After establishing a comprehensive transcriptional picture of the bladder innate immune signature generated in response to FliC, we next examined whether this signature could be exploited for infection control. For this, mice were administered 30 μg FliC into the bladder at 2 h prior to, or 24 h after, infectious challenge with UPEC, and bacterial loads were subsequently determined (24 h later). Mice that received prophylactic FliC had 80% fewer UPEC in the bladder than control mice that received carrier alone (*P* = 0.028) (Fig. 7A). Similarly, mice that received FliC therapeutically exhibited 90% fewer UPEC in the bladder than control mice (*P* = 0.039) (Fig. 7B). There were no significant differences in the numbers of UPEC in urine or kidneys of mice between the FliC treatment groups and carrier control groups (Fig. S7). Of note, mice treated prophylactically with the carrier control exhibited significantly more UPEC in urine and kidneys than mice treated therapeutically with carrier control (similar trends were noted for mice treated with FliC) (Fig. S7). Taken together, these data provide experimental evidence that the immune regulatory activity induced by FliC in the bladder can be harnessed to enhance the ability of the host to control UPEC locally in the context of both pre- and postexposure to FliC.

10.1128/mSphere.00545-19.7 FIG S7 UPEC loads in urine samples and kidneys of mice in FliC treatment study. (A) Prophylactic FliC was administered to the bladders of mice at 2 h prior to infectious challenge with UPEC. (B) Therapeutic FliC was administered to the bladders of mice at 24 h after infectious challenge with UPEC. Bacterial loads were determined for all mice at 24 h after infectious challenge. Neither prophylactic nor therapeutic FliC significantly affected the numbers of UPEC in the urine samples or kidneys of mice. Data shown represent pooled data from 2 to 3 independent experiments, each comprising 8 to 10 mice per group (total *n* = 20 to 30 per group). Mann-Whitney U test was performed (data did not satisfy Gaussian distribution or normality tests). ns, not significant. Download FIG S7, PDF file, 0.1 MB. Copyright © 2019 Acharya et al. 2019 Acharya et al. This content is distributed under the terms of the Creative Commons Attribution 4.0 International license.

## DISCUSSION

This study was aimed at defining whether UPEC flagella, typically associated with motility and bacterial adherence, are sensed by the bladder innate immune system as part of a defense strategy utilizing IL-10 to control infection (8). The principle finding is that detection of the major flagellar filament of UPEC, FliC, within the bladder causes a very rapid local response, resulting in IL-10 synthesis. This study also shows that the bladder IL-10 response induced by FliC is contingent upon signaling through TLR5; this study does not show that IL-10 induction is the main effect of FliC but rather that high-resolution mapping of the FliC-responsive bladder transcriptome provides new, comprehensive details of how IL-10 is part of a broader bladder response to the major flagellar filament protein. Combined with the diversity of UPEC strains (of defined phenotypes related to flagella) and *in vitro* models of UTI used in this study, we suggest rapid IL-10 induction in the bladder response to FliC forms part of a TLR5-dependent program within a complex innate host defense strategy initiated to combat UPEC.

In aligning this study with prior studies, several links between IL-10 induction and flagella are of note. For example, flagellum components of *Salmonella* and *Yersinia* have been shown to modify IL-10 production. *Salmonella* flagella trigger IL-10 secretion in splenocytes (60), monocytes (23, 61), and serum (62), but the type of host response may depend on the nature of antigen presentation (63). Flagella of *Yersinia* have been shown to induce IL-10 in macrophages (24). Interestingly, however, as part of a *Paracoccidioides* vaccine construct, *Salmonella* FliC inhibited IL-10 production in the lungs of mice (64). The effects of flagella on synthesis of cytokines such as IL-6 have been associated with TLR5 (44, 45). In prior studies, we demonstrated the source of IL-10 in UPEC-infected human urothelial cell-monocyte cocultures is monocytes (not epithelial cells) (8, 10); however, by providing insight into the role of TLR5 in FliC-driven IL-10 responses in UPEC UTI *in vivo*, the current study provides a new understanding of the mechanism underlying this rapid bladder defense response triggered by UPEC. We suggest this is relevant to UPEC UTI in humans, because some individuals harbor a stop codon within the TLR5 open reading frame that is predicted to ablate host responses to flagella (65), and a TLR5 C1174T single-nucleotide polymorphism has been associated with recurrent UTI in adult women (66).

We used enriched E. coli flagellum protein preparations to initially study IL-10 responses in human cell cultures exposed to liquid- or soft-agar-grown E. coli harboring *flhDC* to drive hyperflagellated E. coli or E. coli deficient in *fliC*. The combinations of challenge conditions tested, and analysis of different UPEC strains in addition to MC4100, indicate that IL-10 responses to E. coli flagella are not limited to CFT073. Differences in environmental, growth, and stress conditions, or cross talk mechanisms, might affect flagellar expression differently in distinct E. coli strains (67); however, our analysis of UTI89 and EC958 shows a consistent role for FliC in IL-10 induction in the models tested here. MC4100 may be considered irrelevant to UPEC UTI, but inclusion of non-UPEC E. coli shows that the effects of E. coli flagella on IL-10 are not limited to UPEC. These findings are consistent with previous observations that different flagellum H types (H1, H4, and H7) can induce IL-10 secretion, although H4 flagella was identified as the most potent flagellin type able to induce this cytokine (68). Finally, our data are consistent with the well-established paradigm that TLR5 recognizes FliC monomers, not flagellar filaments, and flagellin-mediated stimulation of cytokine synthesis (including IL-10) occurs in the absence of assembled flagellar filaments (69).

Separate from flagella, other factors in UPEC are likely to contribute to IL-10 responses in the bladder. Our findings based on acellular flagellum stimulation assays and experiments using WT E. coli and *fliC*-deficient strains in cell cultures show levels of IL-10 above the baseline *in vitro* even under conditions where FliC was absent. The main cell types used in the coculture model in this study act in synergy in response to UPEC to promote IL-10 synthesis (10), which is a phenotype not discernible from monocultures (12, 70). Other bacterial factors associated with IL-10 induction are lipopolysaccharide and type III secretion system proteins (71), the latter of which is not relevant to UPEC but is shed from some E. coli strains (enteropathogenic E. coli and enterohemorrhagic E. coli) under some conditions (72). We were careful to remove endotoxin from the treatments used in this study, and the use of pure FliC shows that this factor of UPEC significantly contributes to IL-10 bladder induction. However, it is likely that FliC (and flagella more broadly) is not the sole PAMP of UPEC that triggers IL-10 production in host cells. Other PAMPS of different bacteria may also induce IL-10; for example, peptidoglycan-embedded lipopeptides and cell wall glycopolymers of *Staphylococcus* induce IL-10 in monocytes and macrophages (22). Chlamydial major outer membrane protein triggers the production of IL-10 in macrophages (73). LPS-induced IL-10 production through TLR4 is well described (74–77). Thus, it is likely that additional UPEC factors induce IL-10; however, the current findings are consistent with several prior observations of flagella from *Salmonella* (23, 61) and *Yersinia* (24), which are reported to induce IL-10 in monocytes and macrophages, respectively.

In addition to the effects of UPEC FliC on IL-10 induction, this study defines a multifaceted innate immune response that is engaged in the bladder immediately upon detection of FliC. RNA sequencing identified many factors that have been associated with the host response to flagella in other experimental systems (provided in Table S1 in the supplemental material), illustrating a large degree of consistency in the overall response of the bladder to FliC than other systems. Some factors, such as the genes for serum amyloid A (e.g., *saa1*), that were strongly induced by FliC in this study have been linked to flagellar function previously (78) and may be critical to host defense against UTI (79). In addition, the many novel factors identified to be induced after exposure to FliC in this study, such as multiple predicted genes and genes encoding solute carriers and receptors, have no known links to flagella and will require future investigation into their potential roles in UTI. The transcriptomic data of this study expand our insight into the extent to which the innate immune system is engaged by FliC in the bladder. Several of the responses occurring in the mouse bladder in response to FliC can also be interpreted alongside the responses of human uroepithelial cell-monocyte cocultures to discern numerous consistent responses, such as those for IL-10, IL-1, and IL-6. The complex interplay between IL-10 and the regulation of inflammation in the context of other cytokines, such as IL-6, is reviewed elsewhere (77). Topology analysis identified IL-17 as strongly induced in the mouse bladder response to FliC, consistent with the elevated levels of IL-17 observed in the human cell coculture model of bladder; that IL-17 plays a role in innate defense to UPEC UTI in mice (80); and the findings of the current study implicate FliC in this response. It is likely that several of the cytokines identified as induced by FliC in this study contribute to control of UPEC; for example, other than IL-17, previous studies have shown roles for IL-1β (81), IL-6 (82), and G-CSF (83) in modulating host resistance to UPEC UTI. Taken together, these findings support the idea that IL-10 responses to FliC occur concurrently with a diverse repertoire of antimicrobial products and innate immune mediators that are produced as a result of sensing not only flagellum proteins but also other UPEC cell components.

10.1128/mSphere.00545-19.10 TABLE S1 Observations of transcriptional activity observed in the FliC-responsive bladder transcriptome consistent with host responses to flagella reported in prior studies. Genes are listed with a summary of function and transcriptional activity (fold change and *P* value) in WT mice. Notations are provided for prior studies to reflect protein (P)- or gene (G)-based prior observations. Download Table S1, DOCX file, 0.2 MB. Copyright © 2019 Acharya et al. 2019 Acharya et al. This content is distributed under the terms of the Creative Commons Attribution 4.0 International license.

Several lines of evidence relating to flagellin and TLR5 have been established using studies of Salmonella enterica serotype Typhimurium flagella, probably reflecting in part its abundant peritrichous expression and commercial availability. Most signaling in response to flagellin occurs through TLR5 (44), which relays sensing to cell response networks that drive production of cytokines and chemokines. TLR5 signaling can vary depending on experimental conditions, such as specific tissue or cell location and the type of pathogen (44, 84). Our results show that IL-10 induction in the bladder as part of early defense against UPEC UTI requires TLR5. TLR5-dependent IL-10 secretion has also been described as part of the response to a flagellin fusion protein studied to prevent allergy (85); our findings are consistent with this observation. In the context of UTI, a previous study of mice treated with flagellin by transurethral inoculation showed upregulation of KC (CXCL1), MIP2 (CXCL2), MCP-1 (CCL2), IL-6, and TNF-α in the bladder (86). That study did not investigate *il10*; however, the findings of the current study support the view that TLR5 recognition of flagellin is an important element of the innate immune response to UPEC during the early stages of UTI in mice. It is interesting that Andersen-Nissen et al. (86) found that TLR5-deficient mice are able to control UTI initially with a defect in resistance apparent only after 2 to 5 days postinoculation. Our findings show extensive responses to UPEC FliC within just 2 h; it seems likely this early response (including *il10*) is critical to shape an effective host response that requires additional time to develop and effect restriction of UPEC in the bladder, detectable one or more days later. Flagellin also activates renal collecting duct cells via TLR5, which enables upregulation of CXCL1 and CXCL2 to provide renal host defense against pyelonephritis (87). Additionally, this study describes a novel group of 591 genes that exhibited altered expression (mostly downregulated) in response to FliC independent of TLR5. Identification of this group prompted a search for candidates associated with NLRC4/NAIP activation and IL-1β signaling, because NLRC4 is one of the key inflammasome sensors that responds to bacteria (88); most notably, flagellin from *Salmonella* triggers the pathway following cytosolic recognition of the bacterial ligand, as discussed elsewhere (89). It is NAIP5 and NAIP6, rather than NLRC4, that recognize flagellin (90). Activation of this pathway can lead to NLRC4-mediated pyroptosis and other antimicrobial responses, including shedding of infected epithelial cells and release of prostaglandins and leukotrienes. Among the genes identified as significantly upregulated via TLR5-independent mechanisms following exposure to FliC were those encoding caspase-7 and Gasdermin-D; recently, both of these factors were identified as key substrates downstream of the NLRC4/NAIP5 inflammasome required for resistance to *Legionella* infection (91). Thus, it would be interesting to investigate the role of NLRC4/NAIP5 and associated factors, such as caspase-7 and Gasdermin-D, in resistance to UPEC, particularly in the context of TL5-independent driven responses to flagellin in the host response.

We observed significant downregulation of *tlr5* in WT mice treated with FliC, which is consistent with a previous study that showed treatment with various bacterial ligands downregulated TLR5 expression (92). Other studies have shown responses to flagella in the absence of functional TLR5 signaling (41, 44). TLR11 also forms part of the defense response of the bladder to UPEC in experimental infection in mice (93); we excluded TLR11 from this study because of its absence from the human receptor repertoire and because it has been demonstrated that TLR11 is not a sensor for FliC (52). Further studies are needed to characterize the signaling mechanisms underlying UPEC FliC-mediated and TLR5-dependent IL-10 production. Examples of candidates that would be useful to investigate in characterizing these signaling mechanisms are shown in the TLR signaling KEGG pathway used to interpret these data, which we illustrated with IL-10 highlighted as a notional product of TLR5 engagement (at the time of writing, IL-10 is not included in KEGG pathway 4620). For example, significant upregulation of *myd88*, *nfkb1*, and *nfkb2* suggests these contribute to rapid production of IL-10 through MyD88-dependent mechanisms with quick activation of NF-κB and mitogen-activated protein kinase (MAPK). Other differentially expressed genes, such as those related to macrophage and neutrophil inflammation (e.g., *ccl20–mip-3α* and *ngp*), stress a convergence between canonical TLR signaling and early cellular defense responses to UPEC; others, such as *mrgpra2b* (expressed by neutrophils and mast cells, suggested to have important roles in the innate immune system [94]), *mir-351*, and several predicted genes that were the most strongly upregulated independent of TLR5 (but which have largely uncharacterized functions), underscore the gaps in knowledge of how innate immune responses to UPEC develop and how these might affect the pathogenesis of UTI. Other limitations of this work are the concentrations of FliC used, which are difficult to relate to natural infection; however, similar assay conditions are reported in many published studies on FliC (that have used microgram amounts of less pure FliC); this enables comparison between studies of similar nature.

FliC has been topical in vaccine development for decades and forms part of several recently developed experimental vaccines, including as adjuvant comixed with vaccine antigens and as chimeric or fusion proteins, as reviewed elsewhere (95). For example, an FliC adjuvant has been tested in the context of influenza vaccines in human clinical trials (96). We explored the potential of FliC-driven innate immune responses of the bladder as an approach to infection control of UPEC distinct from vaccine-driven adaptive immunity to gain proof of principle that UPEC FliC is useful for prevention or control of UTI. Our observations of mice administered FliC prophylactically as well as therapeutically provide evidence that FliC is useful for new approaches to the treatment of UTI. In these experiments, higher recovery of UPEC from urine and kidneys of mice treated prophylactically (with carrier or FliC) than from mice treated therapeutically most likely reflects the different time periods used between infectious challenge and UPEC load measurement (i.e., 24 h for prophylactic model versus 48 h for therapeutic model); these differences in recovery of UPEC occurred regardless of the use of FliC; thus, we consider these a reflection of the model rather than effects of FliC. Several flagellum H antigen types have been investigated as part of polyvalent vaccine studies in rats for UPEC (97); however, the problem of flagellin variation and the related need to target multiple virulence factors is an important consideration (98). Finally, the nature of flagellin to shape both innate and adaptive arms of immunity has led to its use as an immunomodulatory antitumor agent (42). How FliC or the immune response to it might be incorporated into novel approaches to treat or prevent UTI remains unclear, but this study establishes that FliC can be used to increase the host’s ability to control UPEC bladder infection. The mechanism of the protective effect observed in this study remains unknown, and addressing the potential role of factors, such as *il10*, would necessitate different models, such as double TLR5^−/−^ and IL-10^−/−^ mice, for example. However, we have no evidence that IL-10 is responsible for the protective effect, and given the many genes and cytokines that are altered in expression after FliC inoculation, future work will need to examine the mechanism by which the observed protective effect from FliC is afforded. Another avenue for analysis could be the use of UPEC FliC as an adjuvant to promote the efficacy of experimental UPEC vaccines, as reported for other pathogens (99), or alternatively, as an immunomodulatory agent; such an approach was shown to activate TLR5 and induce the production of a host defense peptide, BD2, that may boost control of recurrent UPEC UTI (100).

## MATERIALS AND METHODS

### Cell lines and bacteria.

Human 5637 uroepithelial (ATCC number HTB-9), U937 monocyte (ATCC number CRL-1593.2), and mouse J774A.1 (ATCC number TIB-7) cell lines were used. Cells were grown at 37°C with 5% CO_2_ in complete RPMI (cRPMI) medium (RPMI 1640 supplemented with 25 mM HEPES, 2 mM l-glutamine, 10% heat-inactivated fetal bovine serum, 100 mM nonessential amino acids, 1 mM sodium pyruvate, 100 U ml^−1^ penicillin, and 100 mg ml^−1^ streptomycin; Life Technologies, USA).

UPEC reference strains CFT073 (101), UTI89 (102), and EC958 (103) and various derivatives, as well as the commensal E. coli MC4100, were used (Table 1). UPEC derivatives included mutants with targeted deletions in *fliC*, namely, CFT073Δ*fliC* (57), EC958Δ*fliC* (68), and UTI89Δ*fliC* (this study) strains. Additionally, a multiple mutant, termed the CFT073Δ*4* strain, with combined deletions in four major chaperone-usher fimbriae operons (type 1, F1C, and two P fimbrial gene clusters) (113), was used along with its *fliC*-deficient derivative, the CFT073Δ*4* Δ*fliC* strain (57). E. coli MC4100 and UPEC strains carrying isopropyl-β-d-thiogalactopyranoside (IPTG)-inducible p*flhDC* (master operon for flagellar biosynthesis) were used to study hyperflagellated states. Unless otherwise stated, bacteria were grown with agitation (200 rpm) at 37°C in lysogeny broth (LB) or on LB agar (1.5% and 0.25% as required) overnight with antibiotic selection (50 μg/ml kanamycin, 30 μg/ml chloramphenicol) and IPTG (20 mM) as indicated. For motility assays, overnight cultures were prepared in LB broth (with appropriate antibiotics where necessary), and 1 μl of phosphate-buffered saline (PBS) containing approximately 1 × 10^6^ CFU was spotted onto the center of fresh 0.25% LB agar plates (in triplicate) that were supplemented with IPTG and kanamycin as necessary. The plates were incubated at 37°C for 9 h, and rates of motility were determined by measuring the diameter of growth over time. The data are shown as the mean diameter (in millimeters) of motility ± standard errors of the means (SEM) for at least 3 independent experiments.

### Cell coculture and cytokine measurement.

A coculture model of human 5637 uroepithelial cells and U937 monocytes was used for most *in vitro* assays, essentially as described previously (8). Briefly, 1 × 10^5^ uroepithelial cells and 5 × 10^4^ monocytes in cRPMI were seeded together into the wells of a 96-well plate. The cocultures were infected with 1.5 × 10^6^ CFU of UPEC (multiplicity of infection [MOI], 10) and incubated at 37°C with 5% CO_2_ for 5 h, a time point previously associated with IL-10 induction by UPEC *in vitro* (10). For cytokine measurements, supernatants were analyzed using ELISA specific for human IL-10 (number 88-7106-86; eBioscience, USA) or multiplex cytokine assays (Bio-Rad, USA). J774A.1 macrophages were used in parallel assays, as indicated. Cell coculture assays were performed at least three times in independent experiments.

### Preparation of flagellum-enriched E. coli.

Broth cultures of E. coli were grown overnight (10 ml LB) at 37°C with shaking (200 rpm), harvested, and washed in PBS three times (8,000 × *g* for 10 min at 4°C). The cells were adjusted to 3 × 10^7^ CFU/ml in cRPMI medium for use in cocultures. Initially, we tested whether E. coli grown to be flagellum enriched would induce more IL-10 than non-flagellum-enriched E. coli; for this, we used soft-agar cultures to promote swarming growth, which is associated with increased expression of flagellin (105). Soft-agar flagellum-enriched E. coli was prepared using LB agar plates (0.25% agar), onto the surface of which was spotted 10 μl containing 3 × 10^8^ CFU (from overnight LB cultures), as previously described (106). The plates were incubated overnight (37°C), and subsequently areas of hyperflagellated E. coli were excised from the agar, resuspended in 500 μl PBS by pipetting, and centrifuged (1,000 × *g*, 5 min at 4°C) to pellet any residual agar. The supernatants containing the bacteria were then diluted in cRPMI for assay (1.5 × 10^6^ CFU/ml; MOI, 10). Colony counts were performed to determine MOI. Results shown represent at least four independent experiments.

### Preparation of acellular flagella, purification of FliC, and protein analysis.

Protein preparations enriched for flagella were isolated from E. coli using a combination of mechanical shearing and ultracentrifugation, essentially as described elsewhere (107). Briefly, 500-ml cultures were grown with shaking (60 rpm) and washed in PBS, and flagella were sheared using a bead beater (57). The suspensions were centrifuged and the supernatants (with flagella) were filtered (0.45 μm). Bacteria-free flagella were pelleted (135,000 × *g*, 90 min, 4°C) and resuspended in 2 ml PBS for freezing at −20°C. Depolymerization of flagellar filaments into FliC monomers was achieved by heating (60°C, 10 min) prior to analysis, postpurification, or use in downstream assays. FliC was postpurified using fast protein liquid chromatography (FPLC) with an ÄKTA pure protein purification system and a Superdex 200 increase 10/300 GL column (GE Lifesciences) (57). Endotoxin was removed using high-capacity columns (88274; Pierce). Proteins were analyzed using a bicinchoninic acid protein assay kit (number 23227; Thermo Scientific Pierce, USA). Western blots (with anti-flagellum H-pool-E antibody; number 54394; Staten Serum Institut, Denmark) used anti-rabbit IgG horseradish peroxidase conjugate (number sc-2030; Santa Cruz Biotech, USA) and 3,3′-diaminobenzidine substrate. The UPEC FliC proteins prepared in this manner were pure and endotoxin free, as previously described (57).

### Mouse experiments and treatment of bladder.

Examination of the bladder response to flagella and FliC was undertaken using female C57BL/6J or B6.129S1-Tlr5^tm1Flv^/J mice (The Jackson Laboratory, USA, and Animal Resources Centre, Canning Vale, WA) at 10 to 12 weeks of age. Mice were administered approximately 1.5 × 10^8^ to 2.0 × 10^8^ CFU UPEC or 30 μg FliC via the transurethral route in 50 μl PBS at a slow infusion rate (5 μl s^−1^). For the collection of tissues, mice were euthanized by isoflurane anesthesia overdose followed by cervical dislocation. Bladder tissue was collected at 2 h postinoculation, a time point associated with IL-10 responses in UPEC-infected mice (8). For ELISA, bladder was homogenized in a protease inhibitor cocktail (Roche, Castle Hill, NSW, Australia) and clarified at 12,000 × *g* for 20 min at 4°C. Supernatants were stored at –80°C until assay, which was performed using quintuplicate samples in a commercial IL-10 ELISA (Pierce Endogen, Scoresby, VIC, Australia). Independent experiments using groups of five were repeated at least twice.

### RNA isolation, sequencing, and bioinformatics.

For RNA isolation, bladder tissues were homogenized in TRIzol (Life Technologies, Mulgrave, VIC, Australia). RNase-free DNase-treated RNA that passed Bioanalyzer 2100 (Agilent) analysis was used for RNA sequencing. We performed mRNA sequencing on RNA from C57BL/6 and B6.129S1-Tlr5^tm1Flv^/J mice (*n* = 3 to 5 per group) using the Illumina NextSeq 500 platform. Total RNA was subjected to 2 rounds of poly(A)^+^ selection and converted to cDNA. We used TruSeq library generation kits (Illumina, San Diego, California). Library construction consisted of random fragmentation of the poly(A) mRNA, followed by cDNA production using random primers. The ends of the cDNA were repaired and A-tailed, and adaptors were ligated for indexing (with up to 12 different barcodes per lane) during the sequencing runs. The cDNA libraries were quantitated using qPCR in a Roche LightCycler 480 with the Kapa Biosystems kit (Kapa Biosystems, Woburn, Massachusetts) prior to cluster generation. Clusters were generated to yield approximately 725,000 to 825,000 clusters/mm^2^. Cluster density and quality were determined during the run after the first base addition parameters were assessed. We ran paired-end 2× 75-bp sequencing runs to align the cDNA sequences to the reference genome. For data preprocessing and bioinformatics, STAR (version 2.5.3) was used to align the raw RNA sequencing fastq reads to the Gencode GRCm38 p4, release M11, mouse reference genome (108). HTSeq-count, version 0.9.1, was used to estimate transcript abundances (109). DESeq2 then was used to normalize and test for differential expression and regulation. Genes that met certain criteria (i.e., fold change of ≥±2.0, *q* value of <0.05) were accepted as significantly altered in expression (110).

### Control of UPEC in the bladder using FliC.

To explore the potential for FliC and associated innate immune responses in the bladder to be used for infection control or disease prevention purposes, we examined UPEC numbers in the bladders of mice that were treated with 30 μg FliC (in 50 μl PBS) either prophylactically or therapeutically. In the prophylactic model, infectious challenge with UPEC occurred 2 h after administration of FliC, and UPEC titers were measured 24 h after infectious challenge. In the therapeutic model, mice received infectious challenge and, 24 h later, received FliC; 24 h later, UPEC titers were measured. The infectious challenge in both models was 1.5 × 10^8^ to 2.0 × 10^8^ CFU of UPEC CFT073 in 50 μl of PBS inoculated via the transurethral route. Control mice received 50 μl of carrier and were challenged in the same manner. The total bacterial loads in the bladders, urine samples, and kidneys of mice were assessed using standard colony count methods, as previously described elsewhere (8).

### Ethics statement.

This study was carried out in accordance with the national guidelines of the Australian National Health and Medical Research Council. The Institutional Animal Care and Use Committee of the University of Alabama at Birmingham and the Animal Ethics Committee of Griffith University reviewed and approved all animal experimentation protocols used in this study (permits: University of Alabama at Birmingham animal protocol IACUC-10089 and Griffith approval MSC/01/18/AEC).

### Statistics.

Statistical significance was set at a *P* value of ≤0.05. Welch’s independent samples *t* test was used to compare IL-10 levels in ELISAs, and analysis of variance (ANOVA) was performed with Tukey’s *post hoc* comparison for multiple-target Bio-Plex assay. Mann-Whitney U test was used to evaluate mouse bladder titer data. Statistical testing of RNA sequencing data was undertaken using DESeq2 and included significance criteria of a fold change of ≥±2.0 and *q* value of <0.05, as described elsewhere (110). Other statistical analyses were performed using GraphPad Prism v8.0 and SPSS Statistical Package v22.

### Data availability.

Raw and processed data were deposited in Gene Expression Omnibus (GEO; accession no. GSE132294).

## References

[1] Foxman B 2014 Urinary tract infection syndromes: occurrence, recurrence, bacteriology, risk factors, and disease burden. Infect Dis Clin North Am 28:1–13. doi:10.1016/j.idc.2013.09.003. 24484571

[2] Griebling TL 2005 Urologic diseases in America project: trends in resource use for urinary tract infections in men. J Urol 173:1288–1294. doi:10.1097/01.ju.0000155595.98120.8e. 15758784

[3] Bacheller CD, Bernstein JM 1997 Urinary tract infections. Med Clin North Am 81:719–730. doi:10.1016/s0025-7125(05)70542-3. 9167654

[4] Emody L, Kerenyi M, Nagy G 2003 Virulence factors of uropathogenic *Escherichia coli*. Int J Antimicrob Agents 22(Suppl 2):29–33. doi:10.1016/s0924-8579(03)00236-x. 14527768

[5] Totsika M, Moriel DG, Idris A, Rogers BA, Wurpel DJ, Phan MD, Paterson DL, Schembri MA 2012 Uropathogenic *Escherichia coli* mediated urinary tract infection. Curr Drug Targets 13:1386–1399. doi:10.2174/138945012803530206. 22664092

[6] Hannan TJ, Totsika M, Mansfield KJ, Moore KH, Schembri MA, Hultgren SJ 2012 Host-pathogen checkpoints and population bottlenecks in persistent and intracellular uropathogenic *Escherichia coli* bladder infection. FEMS Microbiol Rev 36:616–648. doi:10.1111/j.1574-6976.2012.00339.x. 22404313 PMC3675774

[7] Ulett GC, Totsika M, Schaale K, Carey AJ, Sweet MJ, Schembri MA 2013 Uropathogenic *Escherichia coli* virulence and innate immune responses during urinary tract infection. Curr Opin Microbiol 16:100–107. doi:10.1016/j.mib.2013.01.005. 23403118

[8] Duell BL, Carey AJ, Tan CK, Cui X, Webb RI, Totsika M, Schembri MA, Derrington P, Irving-Rodgers H, Brooks AJ, Cripps AW, Crowley M, Ulett GC 2012 Innate transcriptional networks activated in bladder in response to uropathogenic *Escherichia coli* drive diverse biological pathways and rapid synthesis of IL-10 for defense against bacterial urinary tract infection. J Immunol 188:781–792. doi:10.4049/jimmunol.1101231. 22184725

[9] Sundac L, Dando SJ, Sullivan MJ, Derrington P, Gerrard J, Ulett GC 2016 Protein-based profiling of the immune response to uropathogenic *Escherichia coli* in adult patients immediately following hospital admission for acute cystitis. Pathog Dis 74:ftw062. doi:10.1093/femspd/ftw062. 27354295

[10] Duell BL, Carey AJ, Dando SJ, Schembri MA, Ulett GC 2013 Human bladder uroepithelial cells synergize with monocytes to promote IL-10 synthesis and other cytokine responses to uropathogenic *Escherichia coli*. PLoS One 8:e78013. doi:10.1371/journal.pone.0078013. 24155979 PMC3796480

[11] Chan CY, St John AL, Abraham SN 2013 Mast cell interleukin-10 drives localized tolerance in chronic bladder infection. Immunity 38:349–359. doi:10.1016/j.immuni.2012.10.019. 23415912 PMC3647685

[12] Duell BL, Cripps AW, Schembri MA, Ulett GC 2011 Epithelial cell coculture models for studying infectious diseases: benefits and limitations. J Biomed Biotechnol 2011:852419. doi:10.1155/2011/852419. 22007147 PMC3189631

[13] Couper KN, Blount DG, Riley EM 2008 IL-10: the master regulator of immunity to infection. J Immunol 180:5771–5777. doi:10.4049/jimmunol.180.9.5771. 18424693

[14] Duell BL, Tan CK, Carey AJ, Wu F, Cripps AW, Ulett GC 2012 Recent insights into microbial triggers of interleukin-10 production in the host and the impact on infectious disease pathogenesis. FEMS Immunol Med Microbiol 64:295–313. doi:10.1111/j.1574-695X.2012.00931.x. 22268692

[15] Li MC, He SH 2004 IL-10 and its related cytokines for treatment of inflammatory bowel disease. World J Gastroenterol 10:620–625. doi:10.3748/wjg.v10.i5.620. 14991925 PMC4716896

[16] Kane MM, Mosser DM 2001 The role of IL-10 in promoting disease progression in leishmaniasis. J Immunol 166:1141–1147. doi:10.4049/jimmunol.166.2.1141. 11145695

[17] Mege JL, Meghari S, Honstettre A, Capo C, Raoult D 2006 The two faces of interleukin 10 in human infectious diseases. Lancet Infect Dis 6:557–569. doi:10.1016/S1473-3099(06)70577-1. 16931407

[18] Mosser DM, Zhang X 2008 Interleukin-10: new perspectives on an old cytokine. Immunol Rev 226:205–218. doi:10.1111/j.1600-065X.2008.00706.x. 19161426 PMC2724982

[19] Moore KW, de Waal Malefyt R, Coffman RL, O'Garra A 2001 Interleukin-10 and the interleukin-10 receptor. Annu Rev Immunol 19:683–765. doi:10.1146/annurev.immunol.19.1.683. 11244051

[20] Ouyang W, O'Garra A 2019 IL-10 family cytokines IL-10 and IL-22: from basic science to clinical translation. Immunity 50:871–891. doi:10.1016/j.immuni.2019.03.020. 30995504

[21] Price JD, Schaumburg J, Sandin C, Atkinson JP, Lindahl G, Kemper C 2005 Induction of a regulatory phenotype in human CD4+ T cells by streptococcal M protein. J Immunol 175:677–684. doi:10.4049/jimmunol.175.2.677. 16002662

[22] Frodermann V, Chau TA, Sayedyahossein S, Toth JM, Heinrichs DE, Madrenas J 2011 A modulatory interleukin-10 response to staphylococcal peptidoglycan prevents Th1/Th17 adaptive immunity to *Staphylococcus aureus*. J Infect Dis 204:253–262. doi:10.1093/infdis/jir276. 21673036

[23] Wyant TL, Tanner MK, Sztein MB 1999 *Salmonella typhi* flagella are potent inducers of proinflammatory cytokine secretion by human monocytes. Infect Immun 67:3619–3624. 10377147 10.1128/iai.67.7.3619-3624.1999 PMC116552

[24] McNally A, La Ragione RM, Best A, Manning G, Newell DG 2007 An aflagellate mutant *Yersinia enterocolitica* biotype 1A strain displays altered invasion of epithelial cells, persistence in macrophages, and cytokine secretion profiles in vitro. Microbiology 153:1339–1349. doi:10.1099/mic.0.2006/000919-0. 17464048

[25] Chettri JK, Raida MK, Holten-Andersen L, Kania PW, Buchmann K 2011 PAMP induced expression of immune relevant genes in head kidney leukocytes of rainbow trout (*Oncorhynchus mykiss*). Dev Comp Immunol 35:476–482. doi:10.1016/j.dci.2010.12.001. 21147161

[26] Cruz-Córdova A, Rocha-Ramírez LM, Ochoa SA, González-Pedrajo B, Gónzalez-Pedrajo B, Espinosa N, Eslava C, Hernández-Chiñas U, Mendoza-Hernández G, Rodríguez-Leviz A, Valencia-Mayoral P, Sadowinski-Pine S, Hernández-Castro R, Estrada-García I, Muñoz-Hernández O, Rosas I, Xicohtencatl-Cortes J 2012 Flagella from five *Cronobacter* species induce pro-inflammatory cytokines in macrophage derivatives from human monocytes. PLoS One 7:e52091. doi:10.1371/journal.pone.0052091. 23284883 PMC3528739

[27] Gal-Mor O, Suez J, Elhadad D, Porwollik S, Leshem E, Valinsky L, McClelland M, Schwartz E, Rahav G 2012 Molecular and cellular characterization of a *Salmonella enterica* serovar Paratyphi a outbreak strain and the human immune response to infection. Clin Vaccine Immunol 19:146–156. doi:10.1128/CVI.05468-11. 22190395 PMC3272918

[28] Lacave-Lapalun JV, Benderitter M, Linard C 2013 Flagellin or lipopolysaccharide treatment modified macrophage populations after colorectal radiation of rats. J Pharmacol Exp Ther 346:75–85. doi:10.1124/jpet.113.204040. 23596059

[29] Vicente-Suarez I, Brayer J, Villagra A, Cheng F, Sotomayor EM 2009 TLR5 ligation by flagellin converts tolerogenic dendritic cells into activating antigen-presenting cells that preferentially induce T-helper 1 responses. Immunol Lett 125:114–118. doi:10.1016/j.imlet.2009.06.007. 19555720 PMC2758552

[30] Vicente-Suarez I, Takahashi Y, Cheng F, Horna P, Wang HW, Wang HG, Sotomayor EM 2007 Identification of a novel negative role of flagellin in regulating IL-10 production. Eur J Immunol 37:3164–3175. doi:10.1002/eji.200737306. 17948265

[31] Yang X, Murani E, Ponsuksili S, Wimmers K 2013 Association of TLR5 sequence variants and mRNA level with cytokine transcription in pigs. Immunogenetics 65:125–132. doi:10.1007/s00251-012-0662-9. 23132291

[32] Zgair AK 2012 Flagellin administration protects respiratory tract from *Burkholderia cepacia* infection. J Microbiol Biotechnol 22:907–916. doi:10.4014/jmb.1112.11079. 22580309

[33] Lane MC, Alteri CJ, Smith SN, Mobley HL 2007 Expression of flagella is coincident with uropathogenic *Escherichia coli* ascension to the upper urinary tract. Proc Natl Acad Sci U S A 104:16669–16674. doi:10.1073/pnas.0607898104. 17925449 PMC2034267

[34] Lane MC, Lockatell V, Monterosso G, Lamphier D, Weinert J, Hebel JR, Johnson DE, Mobley HL 2005 Role of motility in the colonization of uropathogenic *Escherichia coli* in the urinary tract. Infect Immun 73:7644–7656. doi:10.1128/IAI.73.11.7644-7656.2005. 16239569 PMC1273871

[35] Pichon C, Héchard C, Du Merle L, Chaudray C, Bonne I, Guadagnini S, Vandewalle A, Le Bouguénec C 2009 Uropathogenic *Escherichia coli* AL511 requires flagellum to enter renal collecting duct cells. Cell Microbiol 11:616–628. doi:10.1111/j.1462-5822.2008.01278.x. 19134121

[36] Wright KJ, Seed PC, Hultgren SJ 2005 Uropathogenic *Escherichia coli* flagella aid in efficient urinary tract colonization. Infect Immun 73:7657–7668. doi:10.1128/IAI.73.11.7657-7668.2005. 16239570 PMC1273872

[37] Mavromatis CH, Bokil NJ, Totsika M, Kakkanat A, Schaale K, Cannistraci CV, Ryu T, Beatson SA, Ulett GC, Schembri MA, Sweet MJ, Ravasi T 2015 The co-transcriptome of uropathogenic *Escherichia coli*-infected mouse macrophages reveals new insights into host-pathogen interactions. Cell Microbiol 17:730–746. doi:10.1111/cmi.12397. 25410299 PMC4950338

[38] Duan Q, Zhou M, Zhu L, Zhu G 2013 Flagella and bacterial pathogenicity. J Basic Microbiol 53:1–8. doi:10.1002/jobm.201100335. 22359233

[39] Hung C, Zhou Y, Pinkner JS, Dodson KW, Crowley JR, Heuser J, Chapman MR, Hadjifrangiskou M, Henderson JP, Hultgren SJ 2013 *Escherichia coli* biofilms have an organized and complex extracellular matrix structure. mBio 4:e00645. doi:10.1128/mBio.00645-13. 24023384 PMC3774191

[40] Zhou M, Yang Y, Chen P, Hu H, Hardwidge PR, Zhu G 2015 More than a locomotive organelle: flagella in *Escherichia coli*. Appl Microbiol Biotechnol 99:8883–8890. doi:10.1007/s00253-015-6946-x. 26346269

[41] Ramos HC, Rumbo M, Sirard JC 2004 Bacterial flagellins: mediators of pathogenicity and host immune responses in mucosa. Trends Microbiol 12:509–517. doi:10.1016/j.tim.2004.09.002. 15488392

[42] Hajam IA, Dar PA, Shahnawaz I, Jaume JC, Lee JH 2017 Bacterial flagellin–a potent immunomodulatory agent. Exp Mol Med 49:e373. doi:10.1038/emm.2017.172. 28860663 PMC5628280

[43] Gewirtz AT 2006 Flag in the crossroads: flagellin modulates innate and adaptive immunity. Curr Opin Gastroenterol 22:8–12. doi:10.1097/01.mog.0000194791.59337.28. 16319670

[44] Hayashi F, Smith KD, Ozinsky A, Hawn TR, Yi EC, Goodlett DR, Eng JK, Akira S, Underhill DM, Aderem A 2001 The innate immune response to bacterial flagellin is mediated by Toll-like receptor 5. Nature 410:1099–1103. doi:10.1038/35074106. 11323673

[45] Andersen-Nissen E, Smith KD, Strobe KL, Barrett SL, Cookson BT, Logan SM, Aderem A 2005 Evasion of Toll-like receptor 5 by flagellated bacteria. Proc Natl Acad Sci U S A 102:9247–9252. doi:10.1073/pnas.0502040102. 15956202 PMC1166605

[46] Miao EA, Andersen-Nissen E, Warren SE, Aderem A 2007 TLR5 and Ipaf: dual sensors of bacterial flagellin in the innate immune system. Semin Immunopathol 29:275–288. doi:10.1007/s00281-007-0078-z. 17690885

[47] Lightfield KL, Persson J, Brubaker SW, Witte CE, von Moltke J, Dunipace EA, Henry T, Sun YH, Cado D, Dietrich WF, Monack DM, Tsolis RM, Vance RE 2008 Critical function for Naip5 in inflammasome activation by a conserved carboxy-terminal domain of flagellin. Nat Immunol 9:1171–1178. doi:10.1038/ni.1646. 18724372 PMC2614210

[48] Hatai H, Lepelley A, Zeng W, Hayden MS, Ghosh S 2016 Toll-like receptor 11 (TLR11) interacts with flagellin and profilin through disparate mechanisms. PLoS One 11:e0148987. doi:10.1371/journal.pone.0148987. 26859749 PMC4747465

[49] Shi Z, Cai Z, Yu J, Zhang T, Zhao S, Smeds E, Zhang Q, Wang F, Zhao C, Fu S, Ghosh S, Zhang D 2012 Toll-like receptor 11 (TLR11) prevents *Salmonella* penetration into the murine Peyer patches. J Biol Chem 287:43417–43423. doi:10.1074/jbc.M112.411009. 23135279 PMC3527929

[50] Mathur R, Oh H, Zhang D, Park SG, Seo J, Koblansky A, Hayden MS, Ghosh S 2012 A mouse model of *Salmonella typhi* infection. Cell 151:590–602. doi:10.1016/j.cell.2012.08.042. 23101627 PMC3500584

[51] Mathur R, Zeng W, Hayden MS, Ghosh S 2016 Mice lacking TLR11 exhibit variable *Salmonella typhi* susceptibility. Cell 164:829–830. doi:10.1016/j.cell.2016.02.020. 26919417

[52] Song J, Wilhelm CL, Wangdi T, Maira-Litran T, Lee S-J, Raetz M, Sturge CR, Mirpuri J, Pei J, Grishin NV, McSorley SJ, Gewirtz AT, Bäumler AJ, Pier GB, Galán JE, Yarovinsky F 2016 Absence of TLR11 in mice does not confer susceptibility to *Salmonella Typhi*. Cell 164:827–828. doi:10.1016/j.cell.2016.02.015. 26919416 PMC4963816

[53] Snyder JA, Haugen BJ, Buckles EL, Lockatell CV, Johnson DE, Donnenberg MS, Welch RA, Mobley HL 2004 Transcriptome of uropathogenic *Escherichia coli* during urinary tract infection. Infect Immun 72:6373–6381. doi:10.1128/IAI.72.11.6373-6381.2004. 15501767 PMC523057

[54] Mulvey MA, Schilling JD, Martinez JJ, Hultgren SJ 2000 Bad bugs and beleaguered bladders: interplay between uropathogenic *Escherichia coli* and innate host defenses. Proc Natl Acad Sci U S A 97:8829–8835. doi:10.1073/pnas.97.16.8829. 10922042 PMC34019

[55] Martinez JJ, Mulvey MA, Schilling JD, Pinkner JS, Hultgren SJ 2000 Type 1 pilus-mediated bacterial invasion of bladder epithelial cells. EMBO J 19:2803–2812. doi:10.1093/emboj/19.12.2803. 10856226 PMC203355

[56] Ferenci T, Zhou Z, Betteridge T, Ren Y, Liu Y, Feng L, Reeves PR, Wang L 2009 Genomic sequencing reveals regulatory mutations and recombinational events in the widely used MC4100 lineage of *Escherichia coli* K-12. J Bacteriol 191:4025–4029. doi:10.1128/JB.00118-09. 19376874 PMC2698400

[57] Acharya D, Sullivan MJ, Duell BL, Eveno T, Schembri MA, Ulett GC 2019 Physical extraction and fast protein liquid chromatography for purifying flagella filament from uropathogenic *Escherichia coli* for immune assay. Front Cell Infect Microbiol 9:118. doi:10.3389/fcimb.2019.00118. 31069177 PMC6491459

[58] Lynn DJ, Winsor GL, Chan C, Richard N, Laird MR, Barsky A, Gardy JL, Roche FM, Chan TH, Shah N, Lo R, Naseer M, Que J, Yau M, Acab M, Tulpan D, Whiteside MD, Chikatamarla A, Mah B, Munzner T, Hokamp K, Hancock RE, Brinkman FS 2008 InnateDB: facilitating systems-level analyses of the mammalian innate immune response. Mol Syst Biol 4:218. doi:10.1038/msb.2008.55. 18766178 PMC2564732

[59] Xia J, Benner MJ, Hancock RE 2014 NetworkAnalyst–integrative approaches for protein-protein interaction network analysis and visual exploration. Nucleic Acids Res 42:W167–W174. doi:10.1093/nar/gku443. 24861621 PMC4086107

[60] Sbrogio-Almeida ME, Mosca T, Massis LM, Abrahamsohn IA, Ferreira LC 2004 Host and bacterial factors affecting induction of immune responses to flagellin expressed by attenuated *Salmonella* vaccine strains. Infect Immun 72:2546–2555. doi:10.1128/iai.72.5.2546-2555.2004. 15102762 PMC387842

[61] Ciacci-Woolwine F, Kucera LS, Richardson SH, Iyer NP, Mizel SB 1997 *Salmonellae* activate tumor necrosis factor alpha production in a human promonocytic cell line via a released polypeptide. Infect Immun 65:4624–4633. 9353043 10.1128/iai.65.11.4624-4633.1997 PMC175664

[62] Eaves-Pyles T, Murthy K, Liaudet L, Virág L, Ross G, Soriano FG, Szabó C, Salzman AL 2001 Flagellin, a novel mediator of *Salmonella*-induced epithelial activation and systemic inflammation: I kappa B alpha degradation, induction of nitric oxide synthase, induction of proinflammatory mediators, and cardiovascular dysfunction. J Immunol 166:1248–1260. doi:10.4049/jimmunol.166.2.1248. 11145708

[63] Cunningham AF, Khan M, Ball J, Toellner KM, Serre K, Mohr E, MacLennan IC 2004 Responses to the soluble flagellar protein FliC are Th2, while those to FliC on Salmonella are Th1. Eur J Immunol 34:2986–2995. doi:10.1002/eji.200425403. 15384042

[64] Braga CJ, Rittner GM, Munoz Henao JE, Teixeira AF, Massis LM, Sbrogio-Almeida ME, Taborda CP, Travassos LR, Ferreira LC 2009 Paracoccidioides brasiliensis vaccine formulations based on the gp43-derived P10 sequence and the Salmonella enterica FliC flagellin. Infect Immun 77:1700–1707. doi:10.1128/IAI.01470-08. 19204092 PMC2663153

[65] Hawn TR, Verbon A, Lettinga KD, Zhao LP, Li SS, Laws RJ, Skerrett SJ, Beutler B, Schroeder L, Nachman A, Ozinsky A, Smith KD, Aderem A 2003 A common dominant TLR5 stop codon polymorphism abolishes flagellin signaling and is associated with susceptibility to Legionnaires’ disease. J Exp Med 198:1563–1572. doi:10.1084/jem.20031220. 14623910 PMC2194120

[66] Hawn TR, Scholes D, Li SS, Wang H, Yang Y, Roberts PL, Stapleton AE, Janer M, Aderem A, Stamm WE, Zhao LP, Hooton TM 2009 Toll-like receptor polymorphisms and susceptibility to urinary tract infections in adult women. PLoS One 4:e5990. doi:10.1371/journal.pone.0005990. 19543401 PMC2696082

[67] Soutourina OA, Bertin PN 2003 Regulation cascade of flagellar expression in Gram-negative bacteria. FEMS Microbiol Rev 27:505–523. doi:10.1016/S0168-6445(03)00064-0. 14550943

[68] Kakkanat A, Totsika M, Schaale K, Duell BL, Lo AW, Phan MD, Moriel DG, Beatson SA, Sweet MJ, Ulett GC, Schembri MA 2015 The role of H4 flagella in *Escherichia coli* ST131 virulence. Sci Rep 5:16149. doi:10.1038/srep16149. 26548325 PMC4637896

[69] Smith KD, Andersen-Nissen E, Hayashi F, Strobe K, Bergman MA, Barrett SL, Cookson BT, Aderem A 2003 Toll-like receptor 5 recognizes a conserved site on flagellin required for protofilament formation and bacterial motility. Nat Immunol 4:1247–1253. doi:10.1038/ni1011. 14625549

[70] Barrila J, Radtke AL, Crabbe A, Sarker SF, Herbst-Kralovetz MM, Ott CM, Nickerson CA 2010 Organotypic 3D cell culture models: using the rotating wall vessel to study host-pathogen interactions. Nat Rev Microbiol 8:791–801. doi:10.1038/nrmicro2423. 20948552

[71] Nagamatsu K, Kuwae A, Konaka T, Nagai S, Yoshida S, Eguchi M, Watanabe M, Mimuro H, Koyasu S, Abe A 2009 *Bordetella* evades the host immune system by inducing IL-10 through a type III effector, BopN. J Exp Med 206:3073–3088. doi:10.1084/jem.20090494. 20008527 PMC2806459

[72] Komoriya K, Shibano N, Higano T, Azuma N, Yamaguchi S, Aizawa SI 1999 Flagellar proteins and type III-exported virulence factors are the predominant proteins secreted into the culture media of *Salmonella typhimurium*. Mol Microbiol 34:767–779. doi:10.1046/j.1365-2958.1999.01639.x. 10564516

[73] Bermudez-Fajardo A, Stark A-K, El-Kadri R, Penichet ML, Hölzle K, Wittenbrink MM, Hölzle L, Oviedo-Orta E 2011 The effect of *Chlamydophila pneumoniae* major outer membrane protein (MOMP) on macrophage and T cell-mediated immune responses. Immunobiology 216:152–163. doi:10.1016/j.imbio.2010.06.004. 20637522

[74] Pengal RA, Ganesan LP, Wei G, Fang H, Ostrowski MC, Tridandapani S 2006 Lipopolysaccharide-induced production of interleukin-10 is promoted by the serine/threonine kinase Akt. Mol Immunol 43:1557–1564. doi:10.1016/j.molimm.2005.09.022. 16263172

[75] van den Bosch MWM, Palsson-Mcdermott E, Johnson DS, O'Neill LAJ 2014 LPS induces the degradation of programmed cell death protein 4 (PDCD4) to release Twist2, activating c-Maf transcription to promote interleukin-10 production. J Biol Chem 289:22980–22990. doi:10.1074/jbc.M114.573089. 24982420 PMC4132798

[76] Sheedy FJ, Palsson-McDermott E, Hennessy EJ, Martin C, O'Leary JJ, Ruan Q, Johnson DS, Chen Y, O'Neill LAJ 2010 Negative regulation of TLR4 via targeting of the proinflammatory tumor suppressor PDCD4 by the microRNA miR-21. Nat Immunol 11:141–147. doi:10.1038/ni.1828. 19946272

[77] Carey AJ, Tan CK, Ulett GC 2012 Infection-induced IL-10 and JAK-STAT: a review of the molecular circuitry controlling immune hyperactivity in response to pathogenic microbes. JAKSTAT 1:159–167. doi:10.4161/jkst.19918. 24058765 PMC3670239

[78] Murdoch CC, Espenschied ST, Matty MA, Mueller O, Tobin DM, Rawls JF 2019 Intestinal serum amyloid A suppresses systemic neutrophil activation and bactericidal activity in response to microbiota colonization. PLoS Pathog 15:e1007381. doi:10.1371/journal.ppat.1007381. 30845179 PMC6405052

[79] Erman A, Lakota K, Mrak-Poljsak K, Blango MG, Krizan-Hergouth V, Mulvey MA, Sodin-Semrl S, Veranic P 2012 Uropathogenic *Escherichia coli* induces serum amyloid a in mice following urinary tract and systemic inoculation. PLoS One 7:e32933. doi:10.1371/journal.pone.0032933. 22427910 PMC3299708

[80] Sivick KE, Schaller MA, Smith SN, Mobley HL 2010 The innate immune response to uropathogenic *Escherichia coli* involves IL-17A in a murine model of urinary tract infection. J Immunol 184:2065–2075. doi:10.4049/jimmunol.0902386. 20083670 PMC2821792

[81] Symington JW, Wang C, Twentyman J, Owusu-Boaitey N, Schwendener R, Nunez G, Schilling JD, Mysorekar IU 2015 ATG16L1 deficiency in macrophages drives clearance of uropathogenic *E. coli* in an IL-1beta-dependent manner. Mucosal Immunol 8:1388–1399. doi:10.1038/mi.2015.7. 25669147 PMC4532666

[82] Ching CB, Gupta S, Li B, Cortado H, Mayne N, Jackson AR, McHugh KM, Becknell B 2018 Interleukin-6/Stat3 signaling has an essential role in the host antimicrobial response to urinary tract infection. Kidney Int 93:1320–1329. doi:10.1016/j.kint.2017.12.006. 29475562 PMC5967986

[83] Ingersoll MA, Kline KA, Nielsen HV, Hultgren SJ 2008 G-CSF induction early in uropathogenic *Escherichia coli* infection of the urinary tract modulates host immunity. Cell Microbiol 10:2568–2578. doi:10.1111/j.1462-5822.2008.01230.x. 18754853 PMC3036167

[84] Akira S, Uematsu S, Takeuchi O 2006 Pathogen recognition and innate immunity. Cell 124:783–801. doi:10.1016/j.cell.2006.02.015. 16497588

[85] Schulke S, Wolfheimer S, Gadermaier G, Wangorsch A, Siebeneicher S, Briza P, Spreitzer I, Schiller D, Loeschner B, Uematsu S, Ryffel B, Akira S, Waibler Z, Vieths S, Toda M, Scheurer S 2014 Prevention of intestinal allergy in mice by rflaA:Ova is associated with enforced antigen processing and TLR5-dependent IL-10 secretion by mDC. PLoS One 9:e87822. doi:10.1371/journal.pone.0087822. 24516564 PMC3917841

[86] Andersen-Nissen E, Hawn TR, Smith KD, Nachman A, Lampano AE, Uematsu S, Akira S, Aderem A 2007 Cutting edge: Tlr5-/- mice are more susceptible to *Escherichia coli* urinary tract infection. J Immunol 178:4717–4720. doi:10.4049/jimmunol.178.8.4717. 17404249

[87] Bens M, Vimont S, Ben Mkaddem S, Chassin C, Goujon JM, Balloy V, Chignard M, Werts C, Vandewalle A 2014 Flagellin/TLR5 signalling activates renal collecting duct cells and facilitates invasion and cellular translocation of uropathogenic *Escherichia coli*. Cell Microbiol 16:1503–1517. doi:10.1111/cmi.12306. 24779433

[88] Yang J, Liu Z, Xiao TS 2017 Post-translational regulation of inflammasomes. Cell Mol Immunol 14:65–79. doi:10.1038/cmi.2016.29. 27345727 PMC5214939

[89] Chen KW, Groß CJ, Sotomayor FV, Stacey KJ, Tschopp J, Sweet MJ, Schroder K 2014 The neutrophil NLRC4 inflammasome selectively promotes IL-1beta maturation without pyroptosis during acute *Salmonella* challenge. Cell Rep 8:570–582. doi:10.1016/j.celrep.2014.06.028. 25043180

[90] Malik A, Kanneganti TD 2017 Inflammasome activation and assembly at a glance. J Cell Sci 130:3955–3963. doi:10.1242/jcs.207365. 29196474 PMC5769591

[91] Goncalves AV, Margolis SR, Quirino GFS, Mascarenhas DPA, Rauch I, Nichols RD, Ansaldo E, Fontana MF, Vance RE, Zamboni DS 2019 Gasdermin-D and caspase-7 are the key caspase-1/8 substrates downstream of the NAIP5/NLRC4 inflammasome required for restriction of *Legionella pneumophila*. PLoS Pathog 15:e1007886. doi:10.1371/journal.ppat.1007886. 31251782 PMC6622555

[92] Feng T, Cong Y, Alexander K, Elson CO 2012 Regulation of Toll-like receptor 5 gene expression and function on mucosal dendritic cells. PLoS One 7:e35918. doi:10.1371/journal.pone.0035918. 22545147 PMC3335826

[93] Zhang D, Zhang G, Hayden MS, Greenblatt MB, Bussey C, Flavell RA, Ghosh S 2004 A toll-like receptor that prevents infection by uropathogenic bacteria. Science 303:1522–1526. doi:10.1126/science.1094351. 15001781

[94] Dwyer DF, Barrett NA, Austen KF, Immunological Genome Project Consortium 2016 Expression profiling of constitutive mast cells reveals a unique identity within the immune system. Nat Immunol 17:878–887. doi:10.1038/ni.3445. 27135604 PMC5045264

[95] Cui B, Liu X, Fang Y, Zhou P, Zhang Y, Wang Y 2018 Flagellin as a vaccine adjuvant. Expert Rev Vaccines 17:335–349. doi:10.1080/14760584.2018.1457443. 29580106

[96] Taylor DN, Treanor JJ, Strout C, Johnson C, Fitzgerald T, Kavita U, Ozer K, Tussey L, Shaw A 2011 Induction of a potent immune response in the elderly using the TLR-5 agonist, flagellin, with a recombinant hemagglutinin influenza-flagellin fusion vaccine (VAX125, STF2.HA1 SI). Vaccine 29:4897–4902. doi:10.1016/j.vaccine.2011.05.001. 21596084

[97] Kruze D, Biro K, Holzbecher K, Andrial M, Bossart W 1992 Protection by a polyvalent vaccine against challenge infection and pyelonephritis. Urol Res 20:177–181. doi:10.1007/bf00296534. 1553795

[98] Brumbaugh AR, Mobley HL 2012 Preventing urinary tract infection: progress toward an effective *Escherichia coli* vaccine. Expert Rev Vaccines 11:663–676. doi:10.1586/erv.12.36. 22873125 PMC3498450

[99] Honko AN, Mizel SB 2005 Effects of flagellin on innate and adaptive immunity. Immunol Res 33:83–101. doi:10.1385/IR:33:1:083. 16120974

[100] Ali ASM, Mowbray C, Lanz M, Stanton A, Bowen S, Varley CL, Hilton P, Brown K, Robson W, Southgate J, Aldridge PD, Tyson-Capper A, Abraham S, Pickard RS, Hall J 2017 Targeting deficiencies in the TLR5 mediated vaginal response to treat female recurrent urinary tract infection. Sci Rep 7:11039. doi:10.1038/s41598-017-10445-4. 28887442 PMC5591273

[101] Mobley HL, Green DM, Trifillis AL, Johnson DE, Chippendale GR, Lockatell CV, Jones BD, Warren JW 1990 Pyelonephritogenic *Escherichia coli* and killing of cultured human renal proximal tubular epithelial cells: role of hemolysin in some strains. Infect Immun 58:1281–1289. 2182540 10.1128/iai.58.5.1281-1289.1990 PMC258621

[102] Mulvey MA, Schilling JD, Hultgren SJ 2001 Establishment of a persistent *Escherichia coli* reservoir during the acute phase of a bladder infection. Infect Immun 69:4572–4579. doi:10.1128/IAI.69.7.4572-4579.2001. 11402001 PMC98534

[103] Totsika M, Beatson SA, Sarkar S, Phan MD, Petty NK, Bachmann N, Szubert M, Sidjabat HE, Paterson DL, Upton M, Schembri MA 2011 Insights into a multidrug resistant *Escherichia coli* pathogen of the globally disseminated ST131 lineage: genome analysis and virulence mechanisms. PLoS One 6:e26578. doi:10.1371/journal.pone.0026578. 22053197 PMC3203889

[104] Datsenko KA, Wanner BL 2000 One-step inactivation of chromosomal genes in *Escherichia coli* K-12 using PCR products. Proc Natl Acad Sci U S A 97:6640–6645. doi:10.1073/pnas.120163297. 10829079 PMC18686

[105] Wang Q, Frye JG, McClelland M, Harshey RM 2004 Gene expression patterns during swarming in *Salmonella typhimurium*: genes specific to surface growth and putative new motility and pathogenicity genes. Mol Microbiol 52:169–187. doi:10.1111/j.1365-2958.2003.03977.x. 15049819

[106] Ulett GC, Webb RI, Schembri MA 2006 Antigen-43-mediated autoaggregation impairs motility in *Escherichia coli*. Microbiology 152:2101–2110. doi:10.1099/mic.0.28607-0. 16804184

[107] Gerhardt P, Murray RGE, Wood WA, Krieg NR 1994 Chapter 4.4.1.1.a. Isolation of cell components–procedure for the isolation of flagellar filaments, p 83 *In* Gerhardt P (ed), Methods for general and molecular bacteriology. ASM Press, Washington, DC.

[108] Dobin A, Davis CA, Schlesinger F, Drenkow J, Zaleski C, Jha S, Batut P, Chaisson M, Gingeras TR 2013 STAR: ultrafast universal RNA-seq aligner. Bioinformatics 29:15–21. doi:10.1093/bioinformatics/bts635. 23104886 PMC3530905

[109] Anders S, Pyl PT, Huber W 2015 HTSeq–a Python framework to work with high-throughput sequencing data. Bioinformatics 31:166–169. doi:10.1093/bioinformatics/btu638. 25260700 PMC4287950

[110] Love MI, Huber W, Anders S 2014 Moderated estimation of fold change and dispersion for RNA-seq data with DESeq2. Genome Biol 15:550. doi:10.1186/s13059-014-0550-8. 25516281 PMC4302049

[111] Peters JE, Thate TE, Craig NL 2003 Definition of the *Escherichia coli* MC4100 genome by use of a DNA array. J Bacteriol 185:2017–2021. doi:10.1128/jb.185.6.2017-2021.2003. 12618467 PMC150127

[112] Givskov M, Eberl L, Christiansen G, Benedik MJ, Molin S 1995 Induction of phospholipase- and flagellar synthesis in *Serratia liquefaciens* is controlled by expression of the flagellar master operon flhD. Mol Microbiol 15:445–454. doi:10.1111/j.1365-2958.1995.tb02258.x. 7783615

[113] Wurpel DJ, Totsika M, Allsopp LP, Hartley-Tassell LE, Day CJ, Peters KM, Sarkar S, Ulett GC, Yang J, Tiralongo J, Strugnell RA, Jennings MP, Schembri MA 2014 F9 fimbriae of uropathogenic *Escherichia coli* are expressed at low temperature and recognise Galbeta1-3GlcNAc-containing glycans. PLoS One 9:e93177. doi:10.1371/journal.pone.0093177. 24671091 PMC3966885

