# Supplementary figures and images for: Rapid Bladder Interleukin-10 Synthesis in Response to Uropathogenic Escherichia coli Is Part of a Defense Strategy Triggered by the Major Bacterial Flagellar Filament FliC and Contingent on TLR5
Source: mSphere. 2019 Nov 27;4(6):e00545-19. doi: 10.1128/mSphere.00545-19 (PMC6881718; doi:10.1128/mSphere.00545-19)

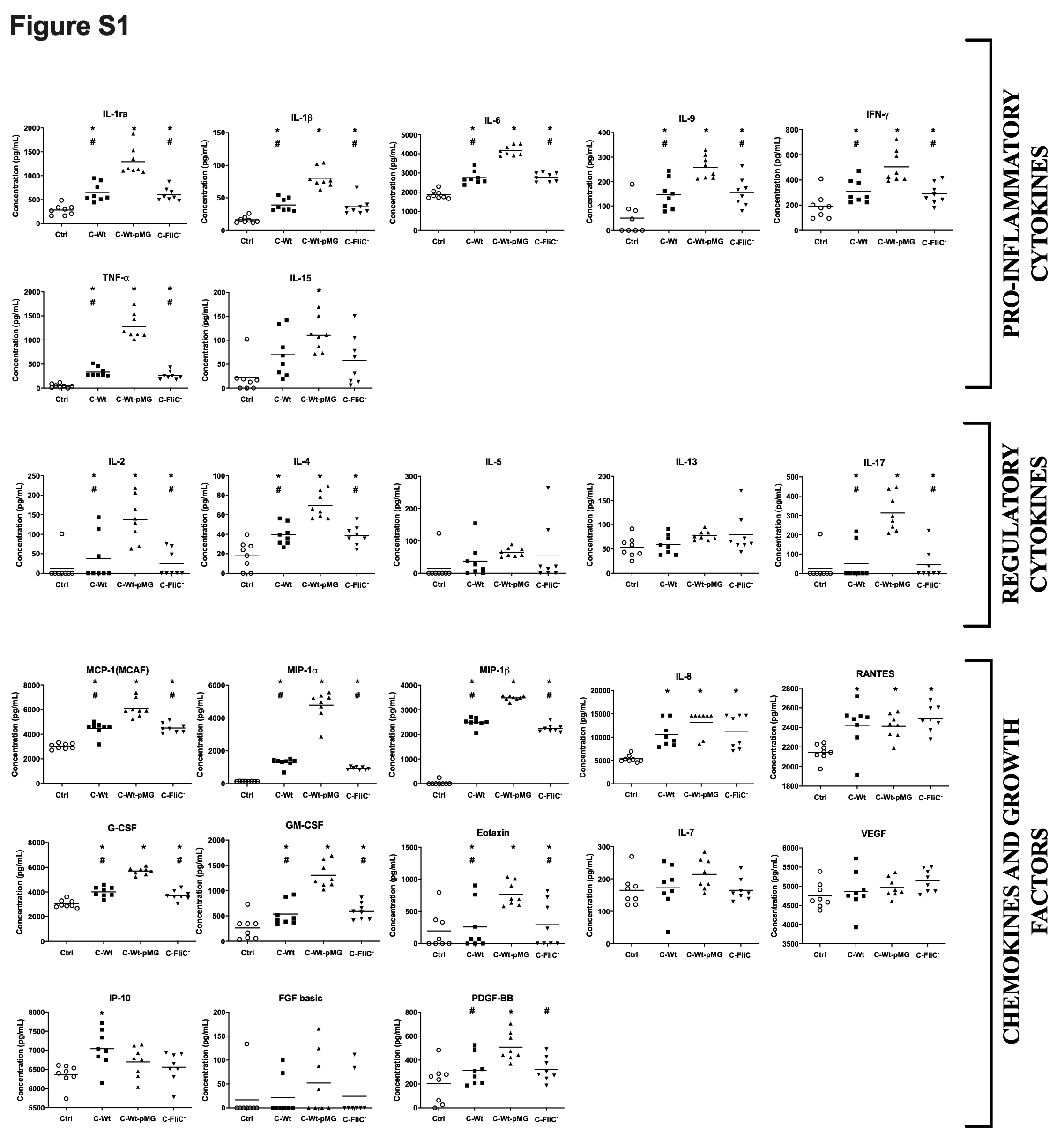

Supplement: FIG S1 [file mSphere.00545-19-sf001.tif]

Figure S2

A

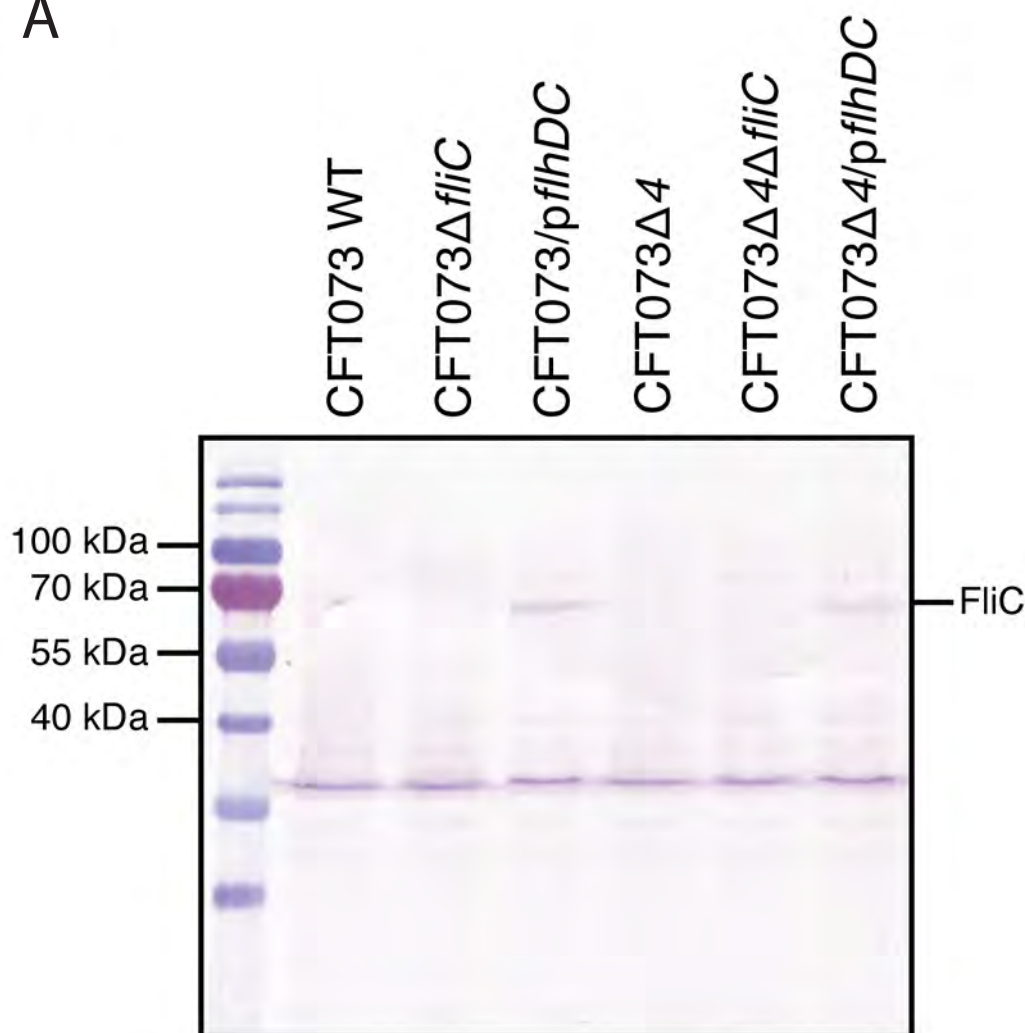

B

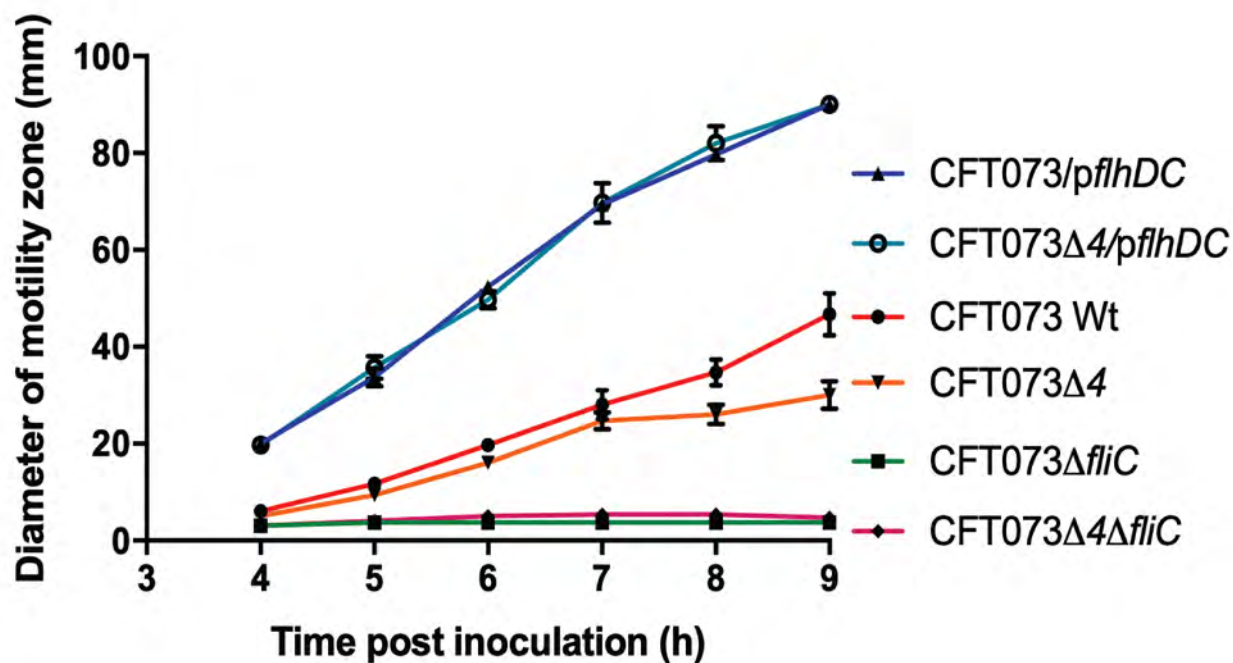

Supplement: FIG S2 [file mSphere.00545-19-sf002.pdf]

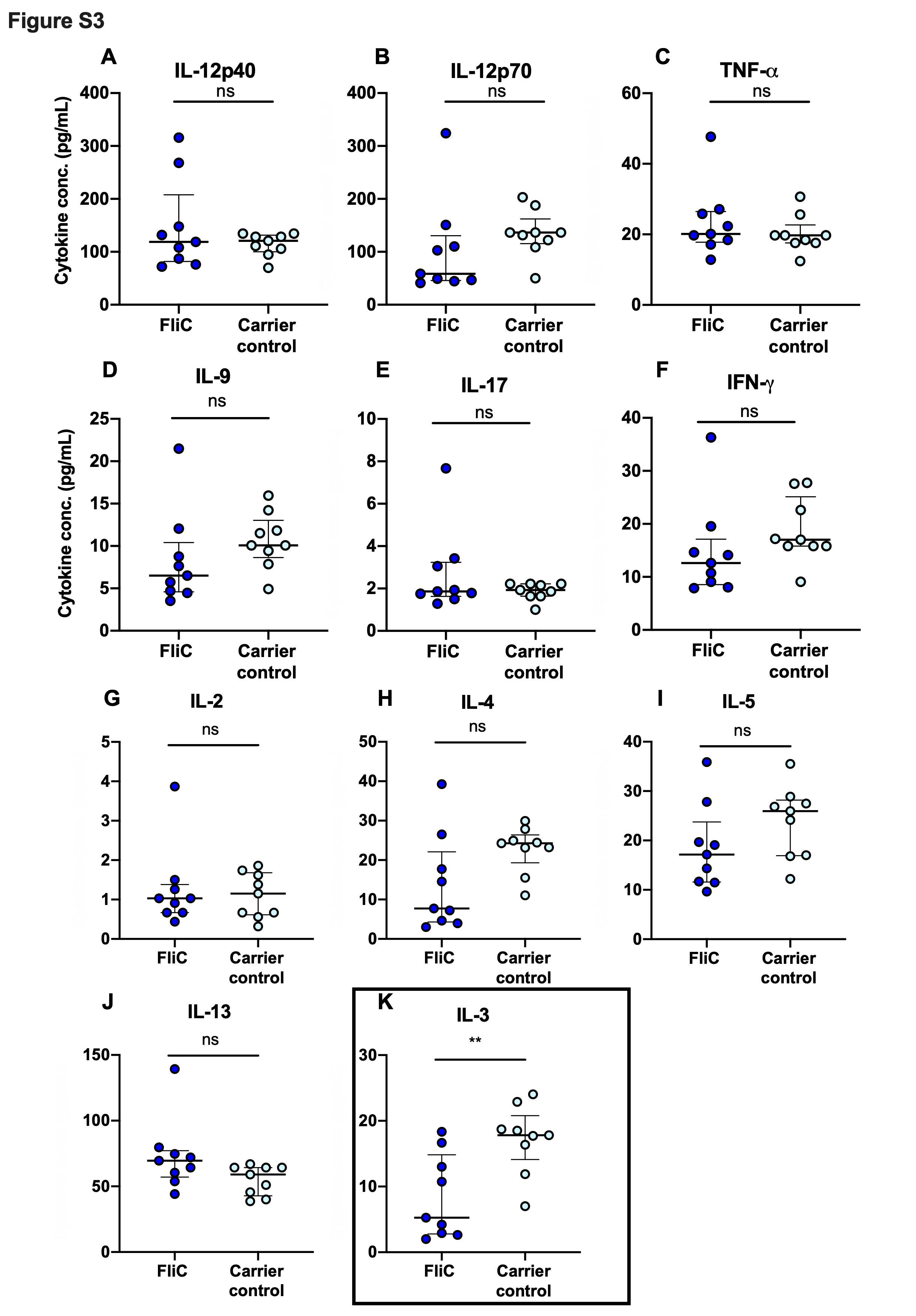

Supplement: FIG S3 [file mSphere.00545-19-sf003.tif]

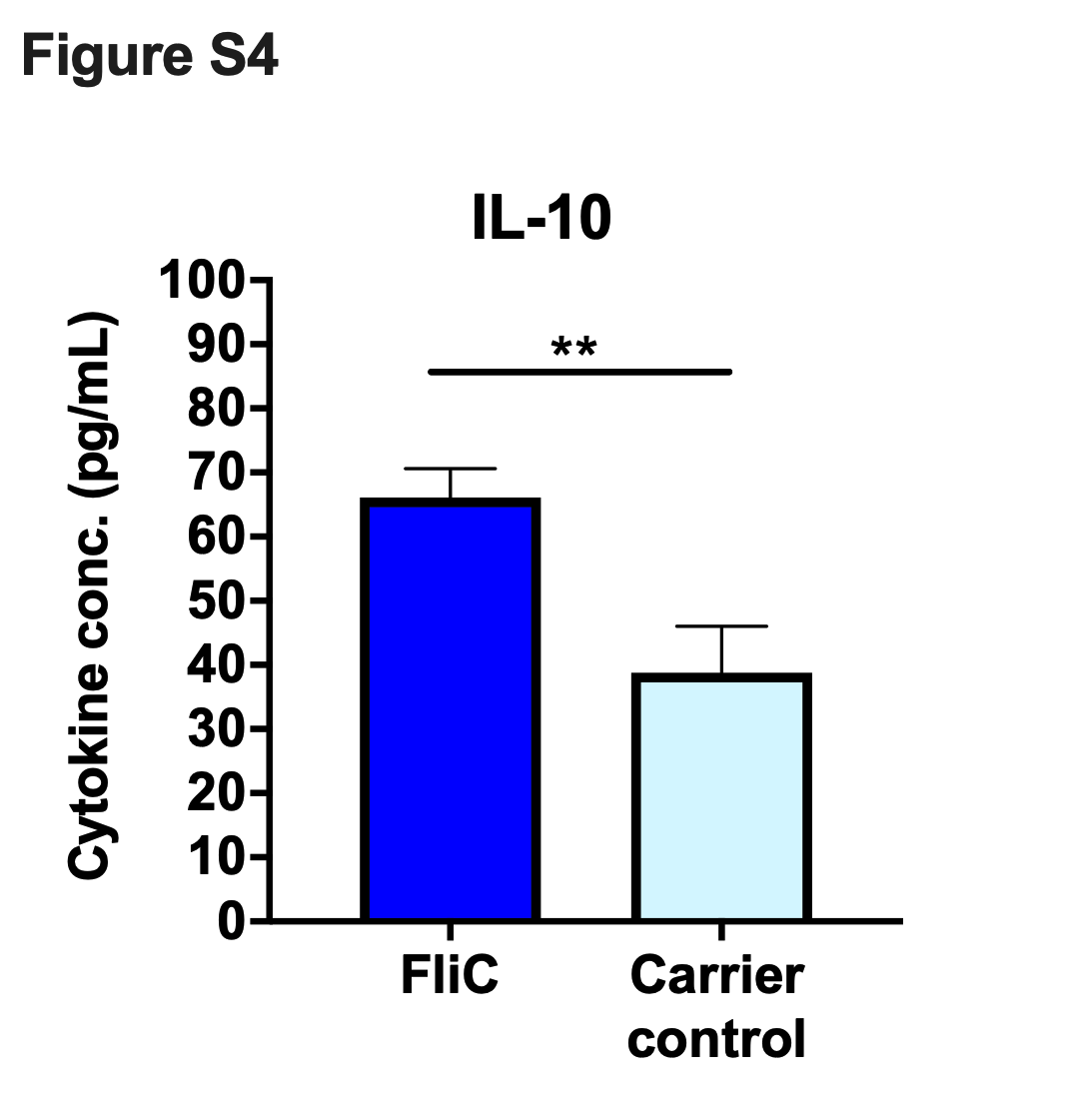

Supplement: FIG S4 [file mSphere.00545-19-sf004.tif]

**Figure S5**

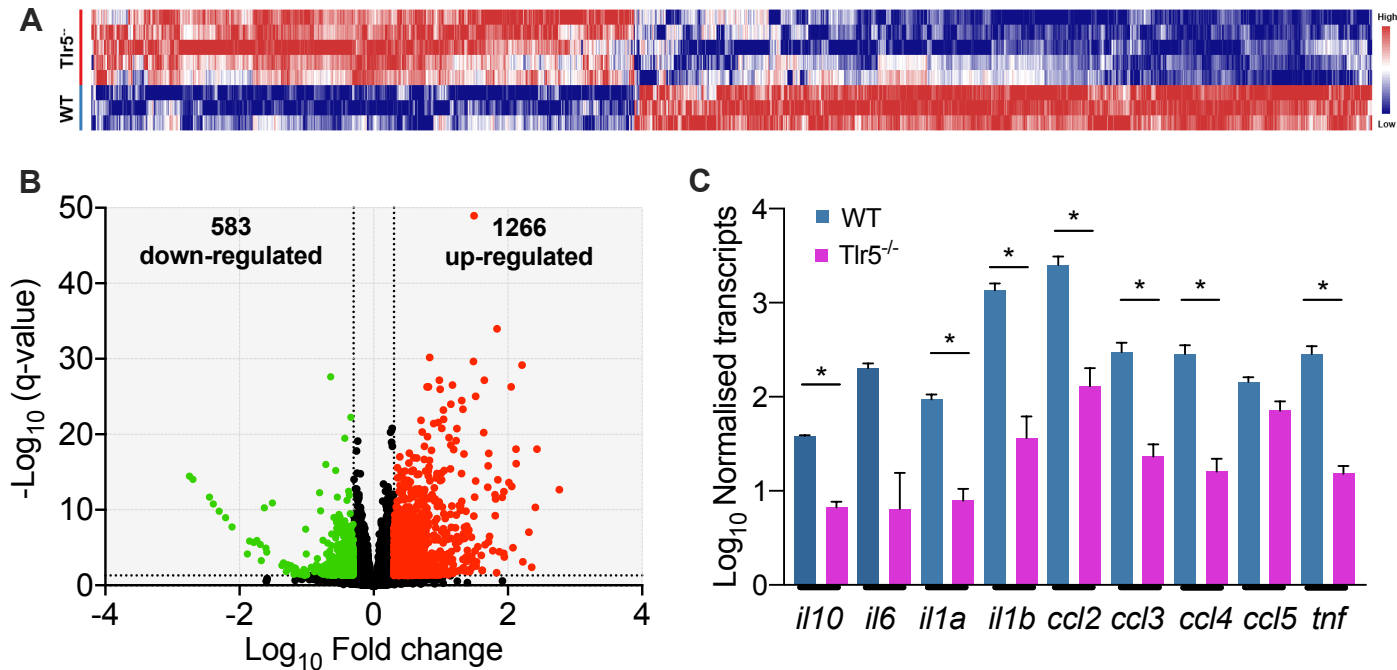

Supplement: FIG S5 [file mSphere.00545-19-sf005.pdf]

# Figure S6

## A

Up-regulated

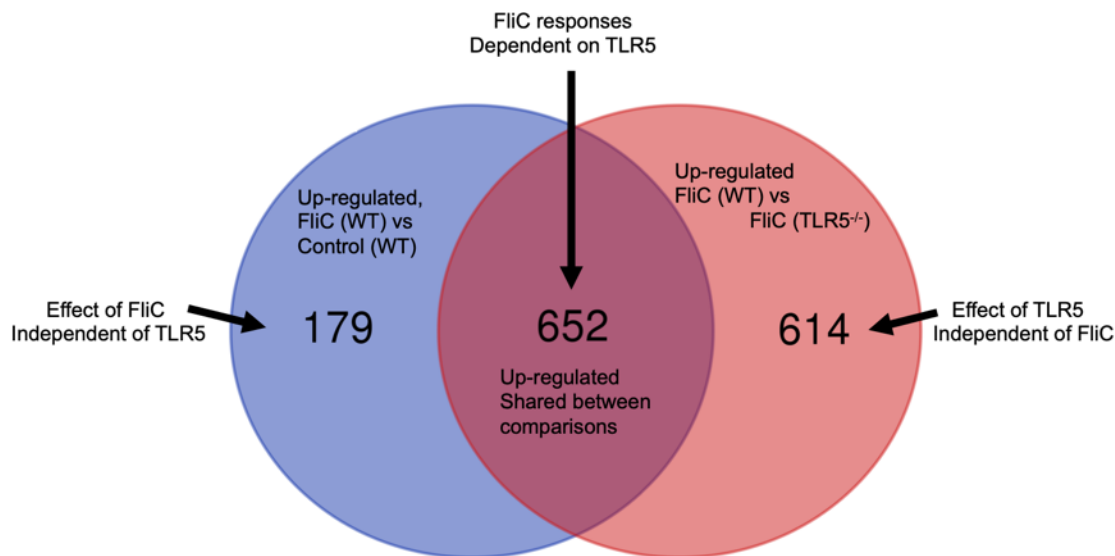

## B

Down-regulated

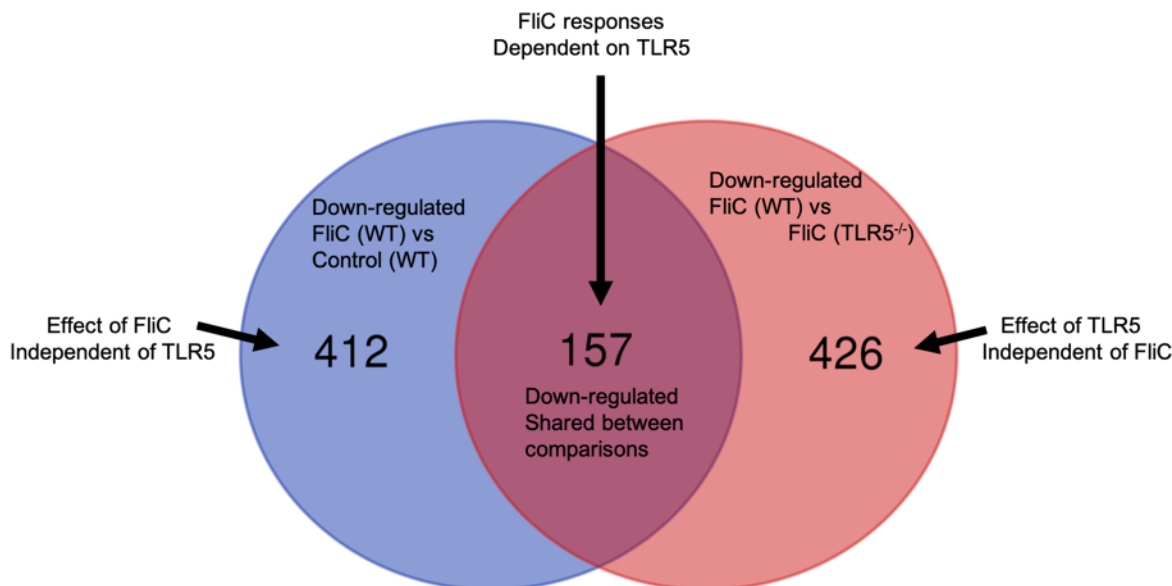

Supplement: FIG S6 [file mSphere.00545-19-sf006.pdf]

**Figure S7**

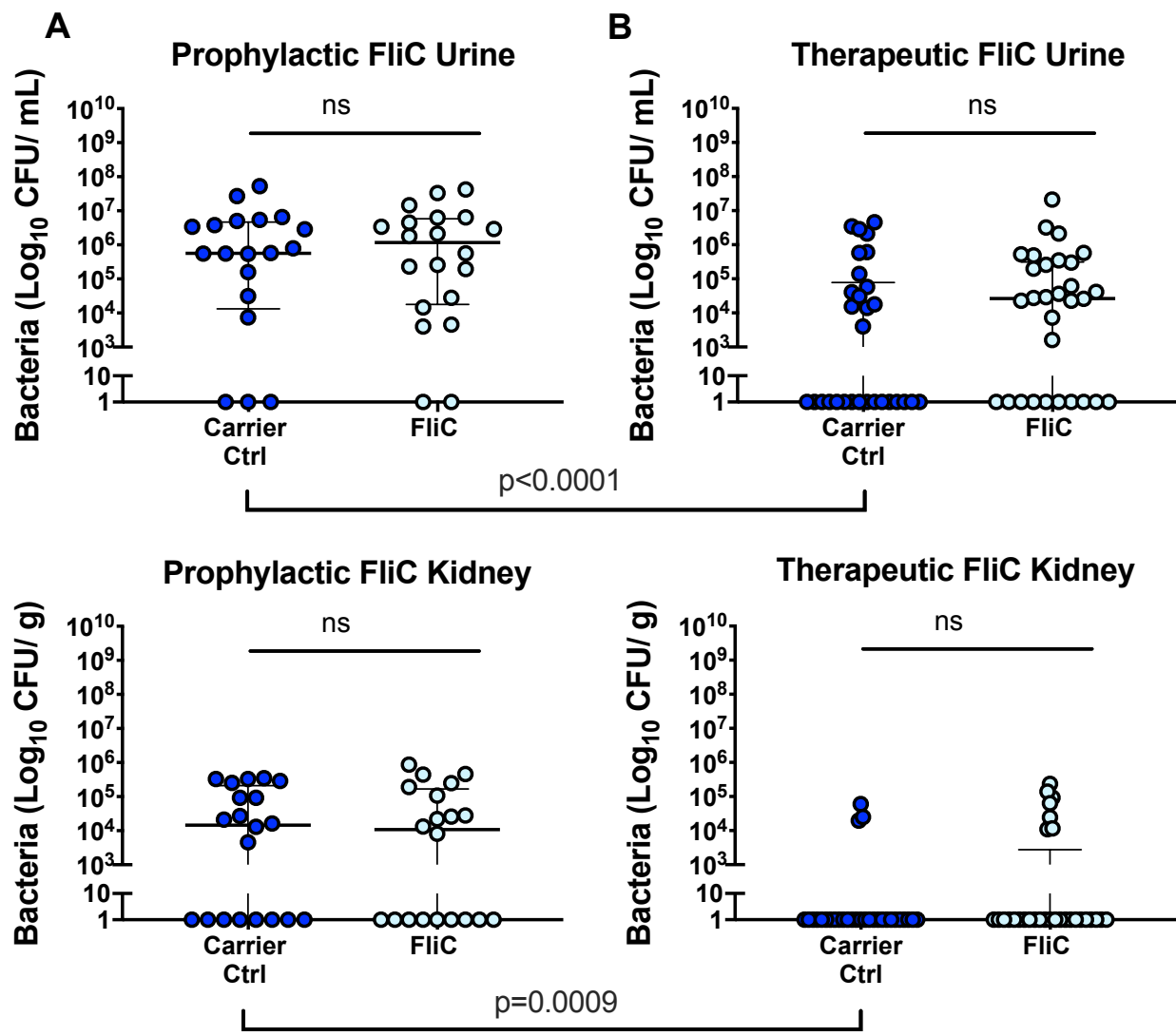

Supplement: FIG S7 [file mSphere.00545-19-sf007.pdf]
