# Supplementary material for: Rapid Bladder Interleukin-10 Synthesis in Response to Uropathogenic Escherichia coli Is Part of a Defense Strategy Triggered by the Major Bacterial Flagellar Filament FliC and Contingent on TLR5
Source: mSphere. 2019 Nov 27;4(6):e00545-19. doi: 10.1128/mSphere.00545-19 (PMC6881718; doi:10.1128/mSphere.00545-19)
Supplement: TABLE S1 [file mSphere.00545-19-st001.docx]

Table S1

| **Gene** | **Summary of Associated Function** | **Fold Change*^a^*** | **P value** | **Protein (P)/**  **Gene (G)** | **Reference** |
| --- | --- | --- | --- | --- | --- |
|  |  | **This study** | | **Previous studies** | |
| **Pro-inflammatory** | | | | | |
| *tnf* | cell signalling protein; activate NF-κB and MAPK pathways | 15.53 | 3.2E-34 | P/G | (1-10) |
| *il1a* | bind to IL-1R and activate TNF-α production | 5.09 | 1.5E-11 | P | (9) |
| *Il1b* | role in cell proliferation, differentiation and apoptosis | 10.91 | 6.8-05 | P/G | (1, 11, 12) |
| *Il6* | role in acute phage response and mediate fever | 3.21 | 0.01 | P/G | (1, 4, 5, 7, 8, 11, 12) |
| *Il12* | T-cell stimulating factor for production of IFN-γ, TNF-α | 1.45 | *ns* | P | (13) |
| *Il23a* | promote inflammation via IL-17 and IL-6 | 2.0 | *ns* | G | (11-15) |
| *Il17c* | induce inflammatory cytokines and chemokines | 29.76 | 8.9E-23 | G | (11, 16) |
| *Il17f* | induce pro-inflammatory cytokine, anti-pathogenic peptide, chemokine secretion | 4.46 | *ns* | G | (11, 17) |
| **Anti-inflammatory/regulatory cytokines** | | | | | |
| *Il10* | downregulate expression of Th1 cells, MHC-II antigen | 3.26 | 0.005 | P | (1, 5, 10, 18, 19) |
| *Il12b* | regulate IL12 function; secreted by macrophages, B-cells | 1.45 | *ns* | P/G | (5, 12, 20) |
| *Nos2* | catalyzes production of NO from L-arginine | 54.42 | 1.3E-70 | P | (5) |
| *Il22* | induce antimicrobial peptides | *nd* | - | G | (11, 21) |
| *Ifn-b* | Antibacterial, antiviral activity | -2.12 | *ns* | P | (22, 23) |
| **Chemokines** | | | | | |
| *Cxcl8* | chemoattractant for granulocytes | *nd* | - | P/G | (9, 10, 15, 24-28) |
| *Ccl3* | chemoattractant for PMN | 11.47 | 6.4E-24 | P | (5, 7), |
| *Cxcl10* | chemoattractant for monocytes and macrophages | 85.33 | 9.9E-159 | G | (11, 15) |
| *Ccl20* | chemoattractant for lymphocyte | 980.27 | 2.5E-53 | G | (11, 15, 24) |
| *Cxcl2* | chemoattractant for PMN | 78.43 | 1.3E-21 | P/G | (7, 8, 11, 15) |
| *Cxcl1* | chemoattractant for PMN | 67.36 | 4.3E-17 | G | (8, 15) |
| *Ccl2* | chemoattractant for monocytes and basophils | 13.97 | 1.8E-08 | G | (8) |
| *Cxcl5* | epithelial-derived neutrophil-activating peptide 78 | 63.70 | 1.5E-60 | G | (15) |
| *Ccl5* | chemoattractant for T cells, eosinophil and basophil | 2.60 | 0.0001 | P/G | (9, 15) |
| **Growth/remodelling factor** | | | | | |
| *Csf3* | stimulate granulocyte development | 15.31 | 1.8E-23 | P/G | (9, 11) |
| *Mmp13* | breakdown extracellular matrix | 18.03 | 3.0E-28 | G | (11) |
| **Antimicrobial peptides** | | | | | |
| *Reg3g* | binds to bacteria and trigger innate immune response | 23.10 | 5.8E-05 | G | (11, 29, 30) |
| *Reg3b* | binds to peptidoglycan | 11.51 | *ns* | G | (11, 31, 32) |
| *S100a7* | Psoriasin; regulate cell cycle, antimicrobial peptide |  | *ns* | G | (15) |
| *S100a8* | calgranulin A; neutrophil chemotaxis | 75.38 | 2.4E-10 | G | (33, 34) |
| *S100a9* | calgranulin B; neutrophil chemotaxis | 67.82 | 6.5E-07 | G | (11, 33, 34) |
| *Lcn2* | antimicrobial peptide (Lipocalin-2); binds to bacterial siderophores | 91.25 | 1.5E-15 | G | (11, 35) |
| *Hamp* | antimicrobial peptide (Hepcidin); inhibit iron transport | 50.23 | 3.4E-05 | G | (11, 36) |

*nd* (not detected); IL-8 (*cxcl8*) not found in mice (37).

*ns* (not significant).

*^a^* Fold change refers to gene expression in the bladders of Wt mice treated with FliC relative to carrier control.

**Supplemental References**

1. Wyant TL, Tanner MK, Sztein MB. 1999. *Salmonella typhi* flagella are potent inducers of proinflammatory cytokine secretion by human monocytes. Infect Immun 67:3619-24.

2. Ciacci-Woolwine F, Kucera LS, Richardson SH, Iyer NP, Mizel SB. 1997. *Salmonellae* activate tumor necrosis factor alpha production in a human promonocytic cell line via a released polypeptide. Infect Immun 65:4624-33.

3. McDermott PF, Ciacci-Woolwine F, Snipes JA, Mizel SB. 2000. High-affinity interaction between gram-negative flagellin and a cell surface polypeptide results in human monocyte activation. Infect Immun 68:5525-9.

4. Hayashi F, Smith KD, Ozinsky A, Hawn TR, Yi EC, Goodlett DR, Eng JK, Akira S, Underhill DM, Aderem A. 2001. The innate immune response to bacterial flagellin is mediated by Toll-like receptor 5. Nature 410:1099-103.

5. Eaves-Pyles T, Murthy K, Liaudet L, Virag L, Ross G, Soriano FG, Szabo C, Salzman AL. 2001. Flagellin, a novel mediator of *Salmonella*-induced epithelial activation and systemic inflammation: I kappa B alpha degradation, induction of nitric oxide synthase, induction of proinflammatory mediators, and cardiovascular dysfunction. J Immunol 166:1248-60.

6. Moors MA, Li L, Mizel SB. 2001. Activation of interleukin-1 receptor-associated kinase by gram-negative flagellin. Infect Immun 69:4424-9.

7. Honko AN, Mizel SB. 2004. Mucosal administration of flagellin induces innate immunity in the mouse lung. Infect Immun 72:6676-9.

8. Andersen-Nissen E, Hawn TR, Smith KD, Nachman A, Lampano AE, Uematsu S, Akira S, Aderem A. 2007. Cutting edge: Tlr5-/- mice are more susceptible to *Escherichia coli* urinary tract infection. J Immunol 178:4717-20.

9. Vijay-Kumar M, Aitken JD, Sanders CJ, Frias A, Sloane VM, Xu J, Neish AS, Rojas M, Gewirtz AT. 2008. Flagellin treatment protects against chemicals, bacteria, viruses, and radiation. J Immunol 180:8280-5.

10. Cruz-Cordova A, Rocha-Ramirez LM, Ochoa SA, Gonzalez-Pedrajo B, Espinosa N, Eslava C, Hernandez-Chinas U, Mendoza-Hernandez G, Rodriguez-Leviz A, Valencia-Mayoral P, Sadowinski-Pine S, Hernandez-Castro R, Estrada-Garcia I, Munoz-Hernandez O, Rosas I, Xicohtencatl-Cortes J. 2012. Flagella from five *Cronobacter* species induce pro-inflammatory cytokines in macrophage derivatives from human monocytes. PLoS ONE 7:e52091.

11. Porte R, Van Maele L, Munoz-Wolf N, Foligne B, Dumoutier L, Tabareau J, Cayet D, Gosset P, Jonckheere N, Van Seuningen I, Chabalgoity JA, Simonet M, Lamkanfi M, Renauld JC, Sirard JC, Carnoy C. 2017. Flagellin-Mediated Protection against Intestinal *Yersinia pseudotuberculosi*s Infection Does Not Require Interleukin-22. Infect Immun 85.

12. Kinnebrew MA, Buffie CG, Diehl GE, Zenewicz LA, Leiner I, Hohl TM, Flavell RA, Littman DR, Pamer EG. 2012. Interleukin 23 production by intestinal CD103(+)CD11b(+) dendritic cells in response to bacterial flagellin enhances mucosal innate immune defense. Immunity 36:276-87.

13. Vicente-Suarez I, Takahashi Y, Cheng F, Horna P, Wang HW, Wang HG, Sotomayor EM. 2007. Identification of a novel negative role of flagellin in regulating IL-10 production. Eur J Immunol 37:3164-75.

14. Yen D, Cheung J, Scheerens H, Poulet F, McClanahan T, McKenzie B, Kleinschek MA, Owyang A, Mattson J, Blumenschein W, Murphy E, Sathe M, Cua DJ, Kastelein RA, Rennick D. 2006. IL-23 is essential for T cell-mediated colitis and promotes inflammation via IL-17 and IL-6. J Clin Invest 116:1310-6.

15. Garcia M, Morello E, Garnier J, Barrault C, Garnier M, Burucoa C, Lecron JC, Si-Tahar M, Bernard FX, Bodet C. 2018. Pseudomonas aeruginosa flagellum is critical for invasion, cutaneous persistence and induction of inflammatory response of skin epidermis. Virulence 9:1163-1175.

16. Chang SH, Reynolds JM, Pappu BP, Chen G, Martinez GJ, Dong C. 2011. Interleukin-17C promotes Th17 cell responses and autoimmune disease via interleukin-17 receptor E. Immunity 35:611-21.

17. Jin W, Dong C. 2013. IL-17 cytokines in immunity and inflammation. Emerging Microbes and Infection 2:e60.

18. Sabharwal N, Chhibber S, Harjai K. 2016. Divalent flagellin immunotherapy provides homologous and heterologous protection in experimental urinary tract infections in mice. Int J Med Microbiol 306:29-37.

19. de Waal Malefyt R, Abrams J, Bennett B, Figdor CG, de Vries JE. 1991. Interleukin 10(IL-10) inhibits cytokine synthesis by human monocytes: an autoregulatory role of IL-10 produced by monocytes. J Exp Med 174:1209-20.

20. Murphy TL, Cleveland MG, Kulesza P, Magram J, Murphy KM. 1995. Regulation of interleukin 12 p40 expression through an NF-kappa B half-site. Mol Cell Biol 15:5258-67.

21. Liang SC, Tan XY, Luxenberg DP, Karim R, Dunussi-Joannopoulos K, Collins M, Fouser LA. 2006. Interleukin (IL)-22 and IL-17 are coexpressed by Th17 cells and cooperatively enhance expression of antimicrobial peptides. J Exp Med 203:2271-9.

22. Mizel SB, Honko AN, Moors MA, Smith PS, West AP. 2003. Induction of macrophage nitric oxide production by Gram-negative flagellin involves signaling via heteromeric Toll-like receptor 5/Toll-like receptor 4 complexes. J Immunol 170:6217-23.

23. Bekisz J, Schmeisser H, Hernandez J, Goldman ND, Zoon KC. 2004. Human interferons alpha, beta and omega. Growth Factors 22:243-51.

24. Sierro F, Dubois B, Coste A, Kaiserlian D, Kraehenbuhl JP, Sirard JC. 2001. Flagellin stimulation of intestinal epithelial cells triggers CCL20-mediated migration of dendritic cells. Proc Natl Acad Sci U S A 98:13722-7.

25. Steiner TS, Nataro JP, Poteet-Smith CE, Smith JA, Guerrant RL. 2000. Enteroaggregative *Escherichia coli* expresses a novel flagellin that causes IL-8 release from intestinal epithelial cells. J Clin Invest 105:1769-77.

26. Gewirtz AT, Navas TA, Lyons S, Godowski PJ, Madara JL. 2001. Cutting edge: bacterial flagellin activates basolaterally expressed TLR5 to induce epithelial proinflammatory gene expression. J Immunol 167:1882-5.

27. Berin MC, Darfeuille-Michaud A, Egan LJ, Miyamoto Y, Kagnoff MF. 2002. Role of EHEC O157:H7 virulence factors in the activation of intestinal epithelial cell NF-kappaB and MAP kinase pathways and the upregulated expression of interleukin 8. Cell Microbiol 4:635-48.

28. Hybiske K, Ichikawa JK, Huang V, Lory SJ, Machen TE. 2004. Cystic fibrosis airway epithelial cell polarity and bacterial flagellin determine host response to *Pseudomonas aeruginosa*. Cell Microbiol 6:49-63.

29. Kinnebrew MA, Ubeda C, Zenewicz LA, Smith N, Flavell RA, Pamer EG. 2010. Bacterial flagellin stimulates Toll-like receptor 5-dependent defense against vancomycin-resistant *Enterococcus* infection. J Infect Dis 201:534-43.

30. Cash HL, Whitham CV, Behrendt CL, Hooper LV. 2006. Symbiotic bacteria direct expression of an intestinal bactericidal lectin. Science 313:1126-30.

31. van Ampting MT, Loonen LM, Schonewille AJ, Konings I, Vink C, Iovanna J, Chamaillard M, Dekker J, van der Meer R, Wells JM, Bovee-Oudenhoven IM. 2012. Intestinally secreted C-type lectin Reg3b attenuates salmonellosis but not listeriosis in mice. Infect Immun 80:1115-20.

32. Dessein R, Gironella M, Vignal C, Peyrin-Biroulet L, Sokol H, Secher T, Lacas-Gervais S, Gratadoux JJ, Lafont F, Dagorn JC, Ryffel B, Akira S, Langella P, Nunez G, Sirard JC, Iovanna J, Simonet M, Chamaillard M. 2009. Toll-like receptor 2 is critical for induction of Reg3 beta expression and intestinal clearance of Yersinia pseudotuberculosis. Gut 58:771-6.

33. Abtin A, Eckhart L, Glaser R, Gmeiner R, Mildner M, Tschachler E. 2010. The antimicrobial heterodimer S100A8/S100A9 (calprotectin) is upregulated by bacterial flagellin in human epidermal keratinocytes. J Invest Dermatol 130:2423-30.

34. Ryckman C, Vandal K, Rouleau P, Talbot M, Tessier PA. 2003. Proinflammatory activities of S100: proteins S100A8, S100A9, and S100A8/A9 induce neutrophil chemotaxis and adhesion. J Immunol 170:3233-42.

35. Flo TH, Smith KD, Sato S, Rodriguez DJ, Holmes MA, Strong RK, Akira S, Aderem A. 2004. Lipocalin 2 mediates an innate immune response to bacterial infection by sequestrating iron. Nature 432:917-21.

36. Rossi E. 2005. Hepcidin--the iron regulatory hormone. Clinical Biochemist Reviews 26:47-9.

37. Sadik CD, Kim ND, Luster AD. 2011. Neutrophils cascading their way to inflammation. Trends Immunol 32:452-60.
